# Supplementary material for: Mice identify subgoal locations through an action-driven mapping process
Source: Neuron. 2023 Jun 21;111(12):1966–1978.e8. doi: 10.1016/j.neuron.2023.03.034 (PMC10636595; doi:10.1016/j.neuron.2023.03.034)
Supplement: Document S1. Figures S1–S5 [file mmc1.pdf]

Neuron, Volume *111*

Supplemental information

Mice identify subgoal locations through an action-driven mapping process

Philip Shamash, Sebastian Lee, Andrew M. Saxe, and Tiago Branco

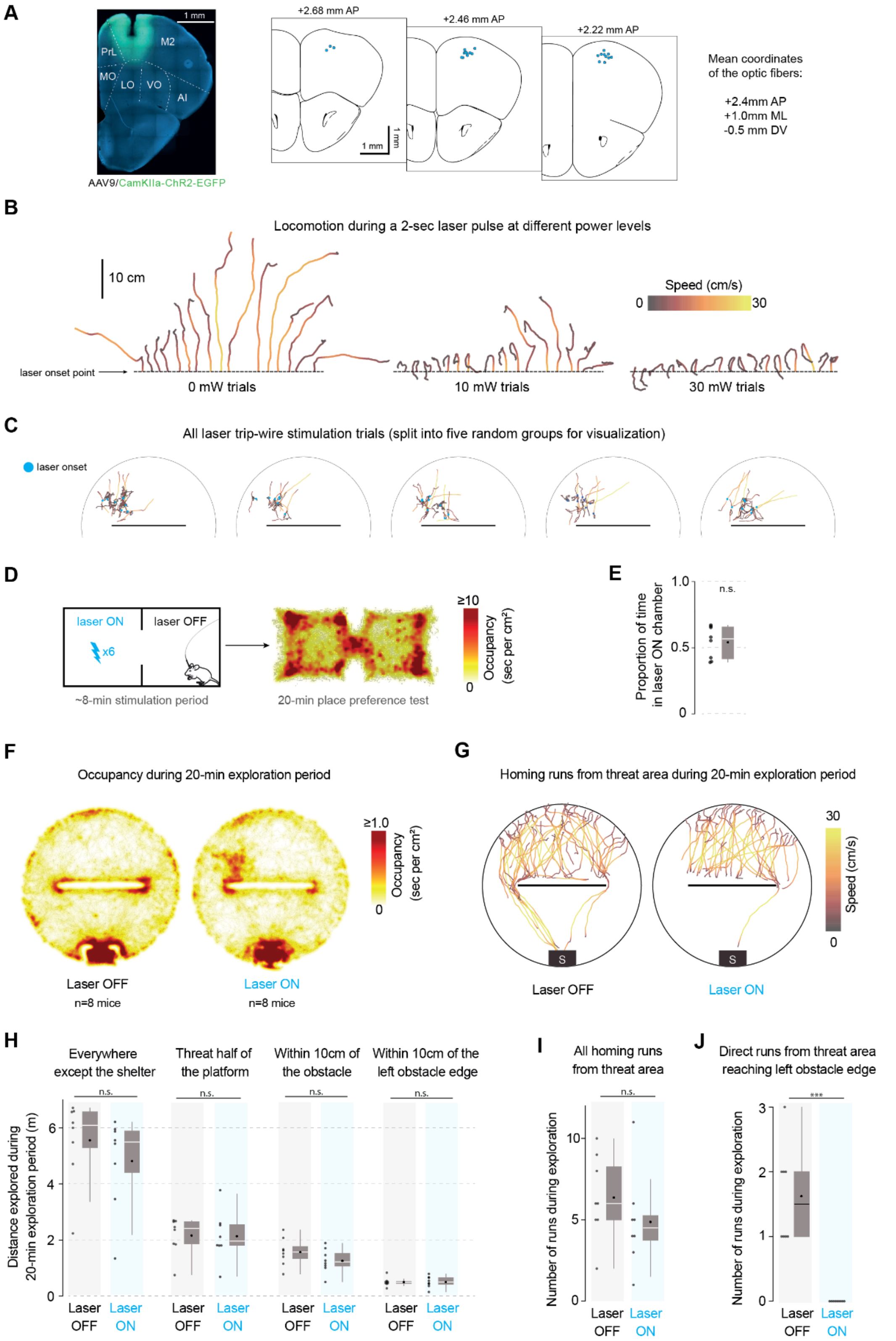

Figure S1: Optogenetic stimulation of right premotor cortex, Related to Figure 1

(A) Left: example viral injection and optic fiber implantation site. M2: supplementary motor cortex (premotor cortex), PrL: prelimbic cortex, MO/LO/VO: medial/lateral/ventral orbital cortex, AI: agranular insular cortex. Right: Putative optic fiber tip locations are overlaid on brain-slice diagrams adapted from [S1] Histological slice images were registered to the Allen Mouse Brain Atlas [S2] using SHARP-Track [S3]to find the fiber tip coordinates. The site of injection with channelrhodopsin was 0.25 mm below (ventral to) the fiber tip. AP and ML coordinates are relative to bregma, and DV coordinates are relative to the brain surface. **(B)** Locomotion following a 2-sec,

20-Hz, 30-mW pulse wave (duty cycle 50%) of 473-nm light in implanted mice. Laser stimulation was triggered manually upon initiation of a running bout, in the behavioral platform with no obstacle and no shelter. Each mouse received 4 trials at each laser power, sequentially interleaved. n = 4 mice. Lines are ordered by the distance and direction of movement following laser onset. **(C)** Trajectories before and after laser stimulation, for the edge-vector blocking protocol. n = 8 mice, 3.5 [2.75,6] (median [IQR]) laser stimulation trials per mouse. **(D)** Place preference assay. Each chamber in the place-preference arena (18 cm x 18 cm x 18 cm) has a distinguishing landmark on the back wall (a large cross and a large circle). After a 1-min habituation period, stimulation consisted of six trials of four repeated 2-sec, 20-Hz, 30-mW pulses (24 total pulses). Stimulation was manually triggered when the mouse fully entered the stimulation side, with at least one minute between trials. The side of stimulation was pseudo-randomly selected such that half of the mice were stimulated on each side. For the occupancy heatmap, stimulation is shown as if it were on the left side for all mice. The heatmap was smoothed with a gaussian filter (σ = 0.3 cm). **(E)** Occupancy in the stimulation chamber is not significantly below 50%. p = 0.7, one-tailed Wilcoxon signed-rank test. n = 8 mice. **(F)** Occupancy heatmaps, smoothed with a gaussian filter (σ = 1 cm). **(G)** Runs from all eight mice in each condition. Left: same runs as in Figure 1a, except with non edge-vector runs also included. Right: Homing runs do not reach the left obstacle edge due to the closed-loop optogenetic stimulation. **(H)** Distance explored is used instead of time explored to account specifically for active exploration. Everywhere except the shelter: p = 0.2; threat half: p = 0.5; obstacle: p = 0.1, edge: p = 0.5, one-tailed permutation tests. **(I)** Total number of homing runs (trajectories shown in panel b): p = 0.15. **(J)** Runs reaching the left edge: $p = 3 \times 10^{-5}$, one-tailed permutation tests.

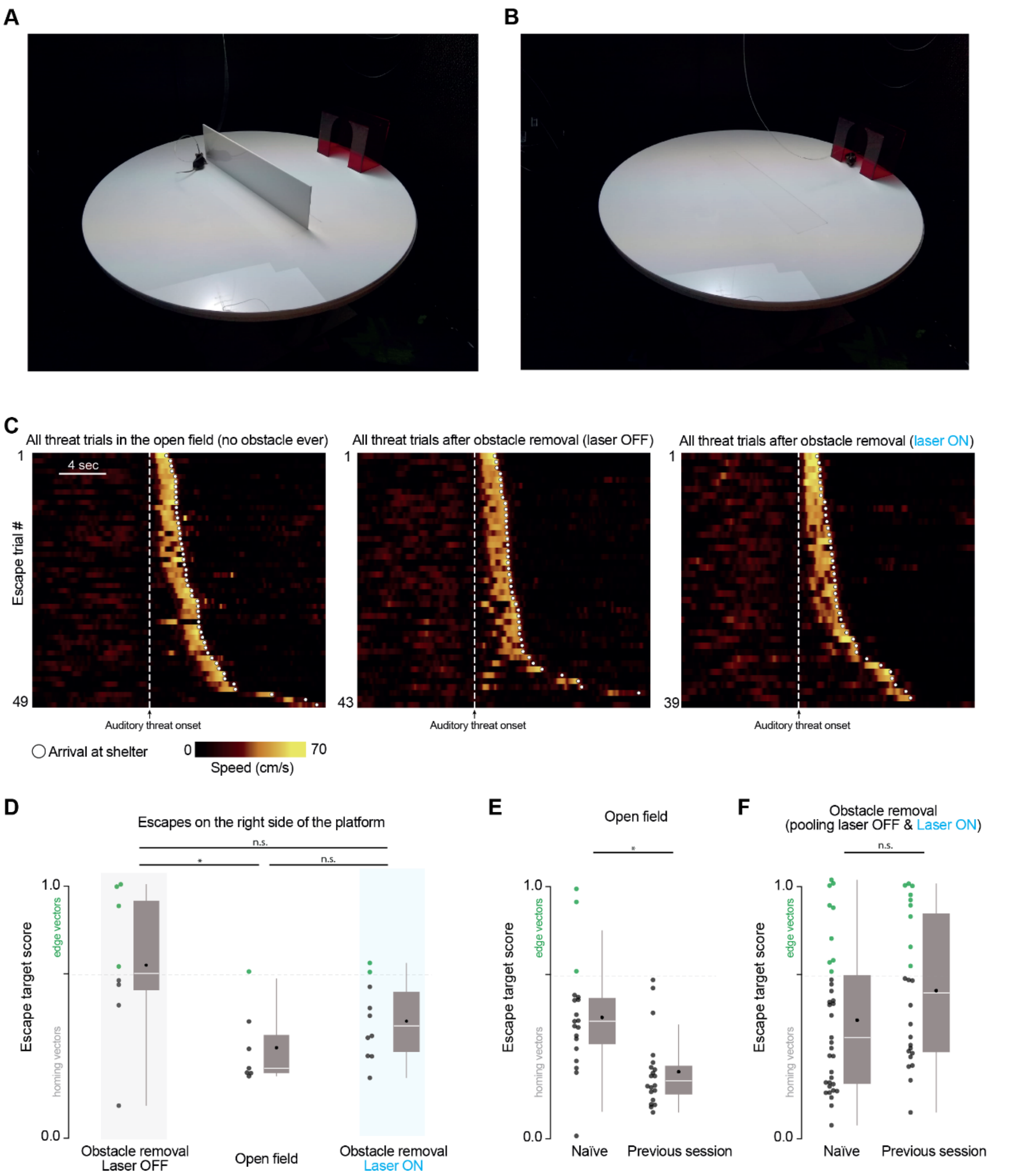

Figure S2: Escape behavior for different conditions, Related to Figure 2

(A) The platform with the wall obstacle. The platform is 92 cm in diameter, and the wall obstacle is 50 cm long x 12.5 cm tall. The shelter is 20 cm wide x 10 cm deep x 15 cm tall. It is made from red acrylic that is opaque to the mouse but transparent to red and infrared light. The mouse has is at the right obstacle edge. **(B)** The platform with no obstacle. A central panel (50 cm wide x 10 cm wide) with the obstacle has been replaced, and a flat panel has been slotted in, in its place. The mouse is sitting in the shelter. **(C)** Open field: n = 8 mice, 29 trials; obstacle removal (laser off): n = 8 mice, 26 trials; obstacle removal (laser on): n = 8 mice, 23 trials. Speed is smoothed with a gaussian filter (σ = 100 ms). **(D)** Escapes on the right side are defined as escapes that, upon passing the center of the arena along the shelter-threat (north-south) axis, were on the right half of the platform. In the main figures, we limited analysis to escapes on the left side of the platform (measured at the center of the shelter-threat axis). This allows us to evaluate our model, in which edge-vector runs generate subgoals one at a time, at the targeted edge. We observed that there were fewer escapes on the right side across all conditions, possibly due to an environmental bias or an effect of the brain implant. As a result, there are not enough data determine whether the laser manipulations has an effect on right-side escapes. Obstacle removal (laser off) vs. open

field: p = .045; Obstacle removal (laser on) vs. open field: p = .1; Obstacle removal (laser off) vs. obstacle removal (laser on): p = .1, one-tailed permutation tests on proportion of edge-vector escapes. Open field: 10 escapes; Obstacle removal (laser off): 8 escapes; Obstacle removal (laser on): 10 escapes. **(E)** In each experiment, 4/8 mice were naive, and 4/8 mice had had a previous behavioral session, in a random condition. Mice with a previous session targeted the shelter more accurately in the open-field environment. P = .03, one-tailed permutation test on proportion of edge-vector escapes. **(F)** Mice with a previous session did not execute significantly more edge-vector escapes than naive mice. p = 0.2, one-tailed permutation test on proportion of edge-vector escapes.

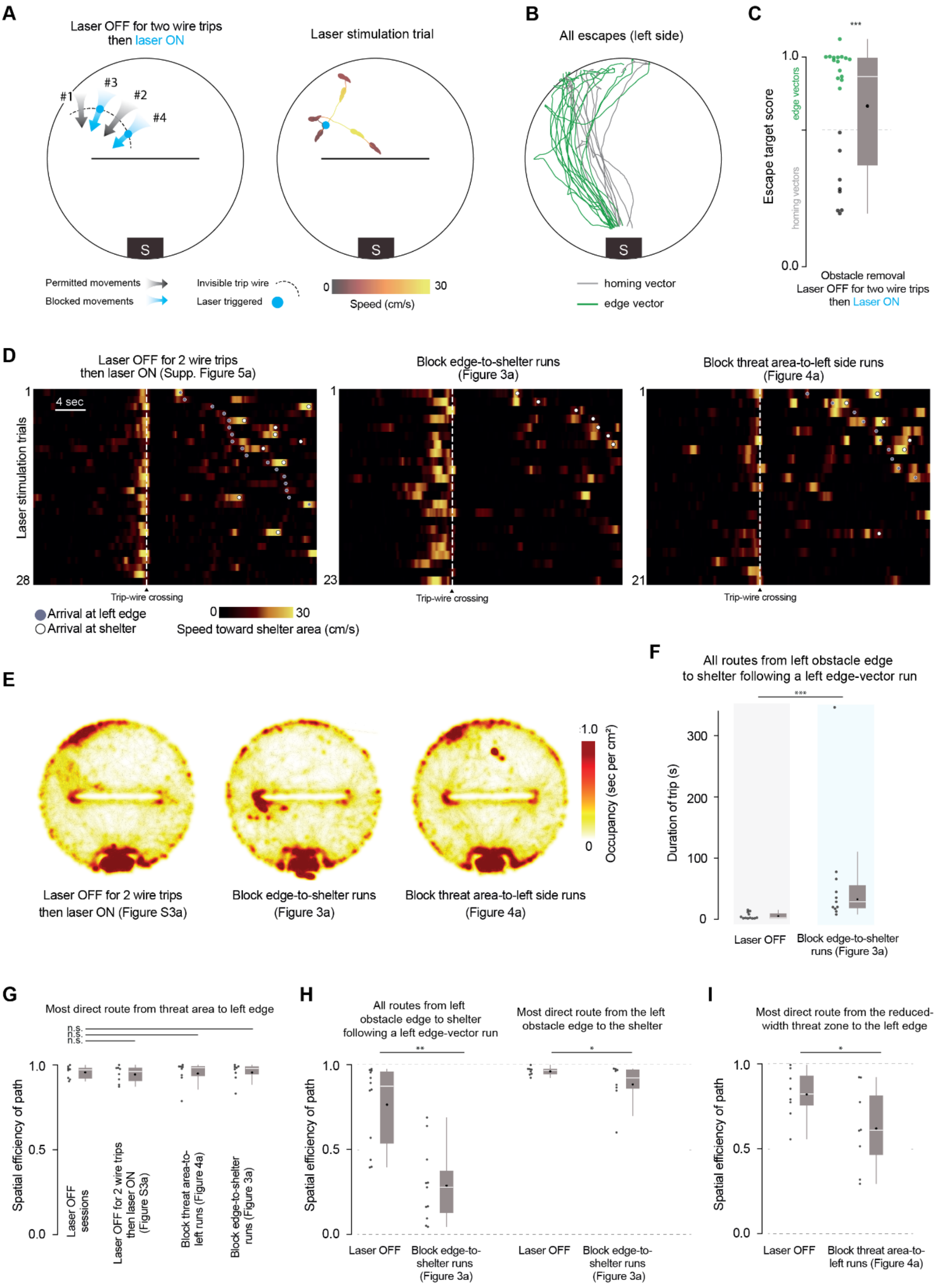

Figure S3: Controls for the effects of optogenetic stimulation, Related to Figure 3

(A) Schematic of stimulation blocking all but the first two edge-vector runs. Stimulation protocol follows the edge-vector-blocking protocol from Figure 1, except that the first two trip-wire crossings are allowed to occur with no laser stimulation. This generated a median of 3.0 laser-stimulation trials per mouse, compared to 3.5 in the original experiment from Figures 1-2. The example shows four seconds after laser onset: the mouse was stimulated for two seconds, and then ran toward the

center of the obstacle. **(B)** Escapes after obstacle removal; n = 8 mice, 23 escapes. **(C)** Obstacle removal (block after two crossings) vs. open field: $p = 3x10^{-4}$ (***); vs. obstacle removal (block edge vectors): p=.003; vs. obstacle removal (laser off): p = .9, one-tailed permutation tests on proportion of edge-vector escapes. **(D)** All trip-wire crossings, with laser stimulation. Left, center: mice must be moving toward the shelter area (i.e., southward) in order to trigger the trip wire. Right: mice must be moving toward the left side in order to trigger the trip wire. **(E)** Occupancy heatmaps are smoothed with a gaussian filter (σ = 1 cm) and overlaid on all movements for all mice (transparent gray dots). There is increased occupancy in the north-west area of the platform in the laser-off-then-on; block threat-to-left-side; and original block-edge-vector conditions due to a pinch of bedding being placed in that area (see Methods). There is increased occupancy near the left obstacle edge in the block edge-to-shelter condition due to the optogenetic stimulation taking place there. **(F)** Each dot represents one trip between the left obstacle edge and the shelter. Laser off median: 2.5 sec; Block edge-to-shelter median: 29 sec. Block edge-to-shelter vs. laser off: p = 0.001, one-tailed permutation test. **(G)** Each dot represents once session (the most efficient path that took place during that session). Laser off-then-on vs. laser off: p = 0.4; block threat-to-left-side vs. laser off: p = 0.3; block edge-to-shelter vs. laser off: p = 0.2, one-tailed permutation tests. **(H)** Left: Each dot represents one trip between the left obstacle edge and the shelter; p = 0.002, one-tailed permutation test. Right: each dot represents one session; p = 0.02, one-tailed permutation test. The effect on the most direct route in the session is relatively weak; this is because, in this experiment, we only blocked edge-to-shelter movements that followed edge-vector runs (i.e., passed the original trip wire from Figure 1). Thus, edge-to-shelter movements that did not pass through the threat area (e.g., running from the shelter to the edge and back) were spared. **(I)** Each dot represents one session; p = 0.02, one-tailed permutation test.

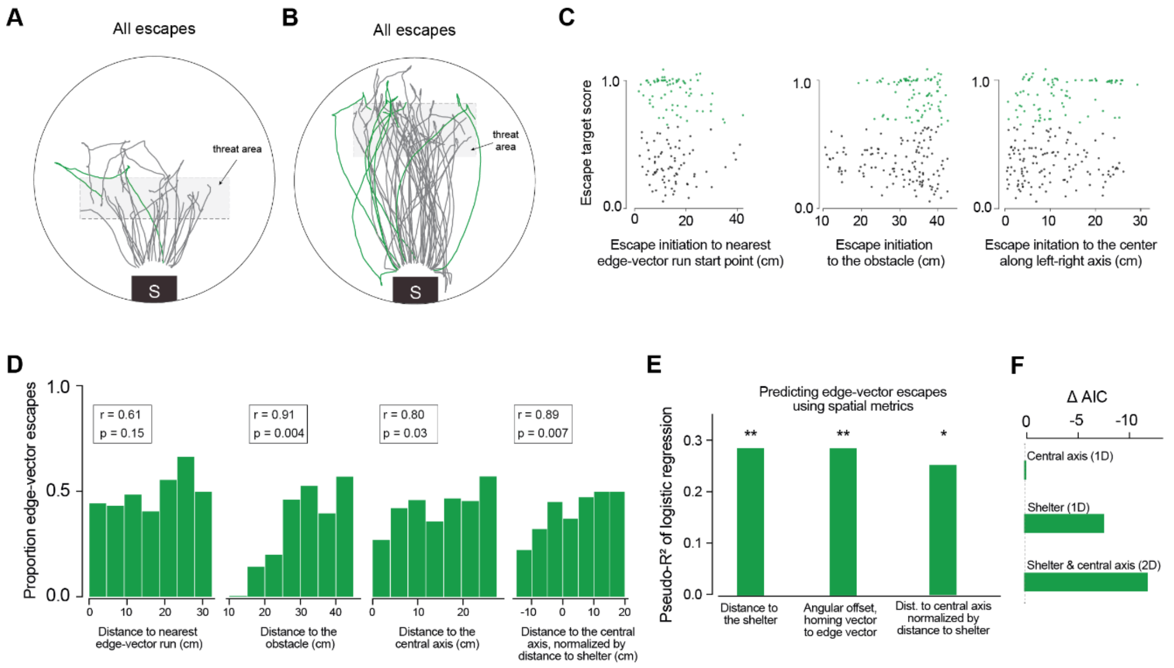

Figure S4: Relationship between escape onset location and subgoal behavior, Related to Figure 4

(A) Escapes triggered after obstacle removal, using the new threat zone, indicated by the red dotted lines. Green trace shows the only escape that begins by moving toward the obstacle edge location (1/28); however, this appears to be a continuation of the pre-threat movement rather than a genuine subgoal escape. **(B)** Escapes triggered after obstacle removal, using another new threat zone, indicated by the red dotted lines. **(C)** Distance metrics plotted against the escape target score. Edge-vector escapes are plotted in green. Distances are measured from the escape initiation point. For the distance to the nearest spontaneous edge-vector run start point (top), only runs toward the same side as the escape are considered. **(D)** To visualize the relationship between position and edge-vector probability, each spatial metric is put into seven equal-sized bins, and the proportion of edge-vector escapes in each bin is taken. All bins have at least six escapes. r-values and p-values come from the Pearson correlation between the spatial bin and the proportion of edge-vector escapes. **(E)** Since the metrics we tested are correlated with each other, we analyzed whether their relationship to escape behavior could all be parsimoniously explained by a sense of distance from the shelter. To test this, we normalized the mouse's distance from the central axis such that it was uncorrelated to the distance from the shelter, using linear regression. This normalized spatial metric retained its capacity to predict edge-vector escapes (pseudo-R^2=0.25; p=0.017, permutation test). This means that, at a given distance from the shelter, mice are more likely to execute edge-vector escapes if they are further from the central axis of the arena McFadden's pseudo-R^2 measures the strength of the relationship between each metric and the odds of executing edge-vector escapes. Distances and angles are measured from the escape initiation point of each escape. For the distance to the nearest spontaneous edge-vector run start point, only runs toward the same side as the escape are considered. Distance to the shelter: pseudo-R^2=0.29; p=0.006. Angular offset between the homing vector and the edge vector: pseudo-R^2=0.29; p=0.006. Distance to the central axis, normalized by the distance to the shelter: pseudo-R^2=0.25; p=0.017. P-values come from a permutation test using 10,000 random shuffles of the edge-vector/homing-vector labels, with the pseudo-R^2 as the test statistic. **(F)** Akaike Information Criterion (AIC) analysis on a logistic regression with different predictors. Decreases in AIC represent better model fit and include a penalty for using additional parameters/predictors (with substantially better fit at δAIC <=2 [S4]). $\delta AIC_i = AIC_i \quad AIC_{m}in$ where $AIC_{m}in$ here is the AIC from the model with the single, distance-from-central-axis predictor.

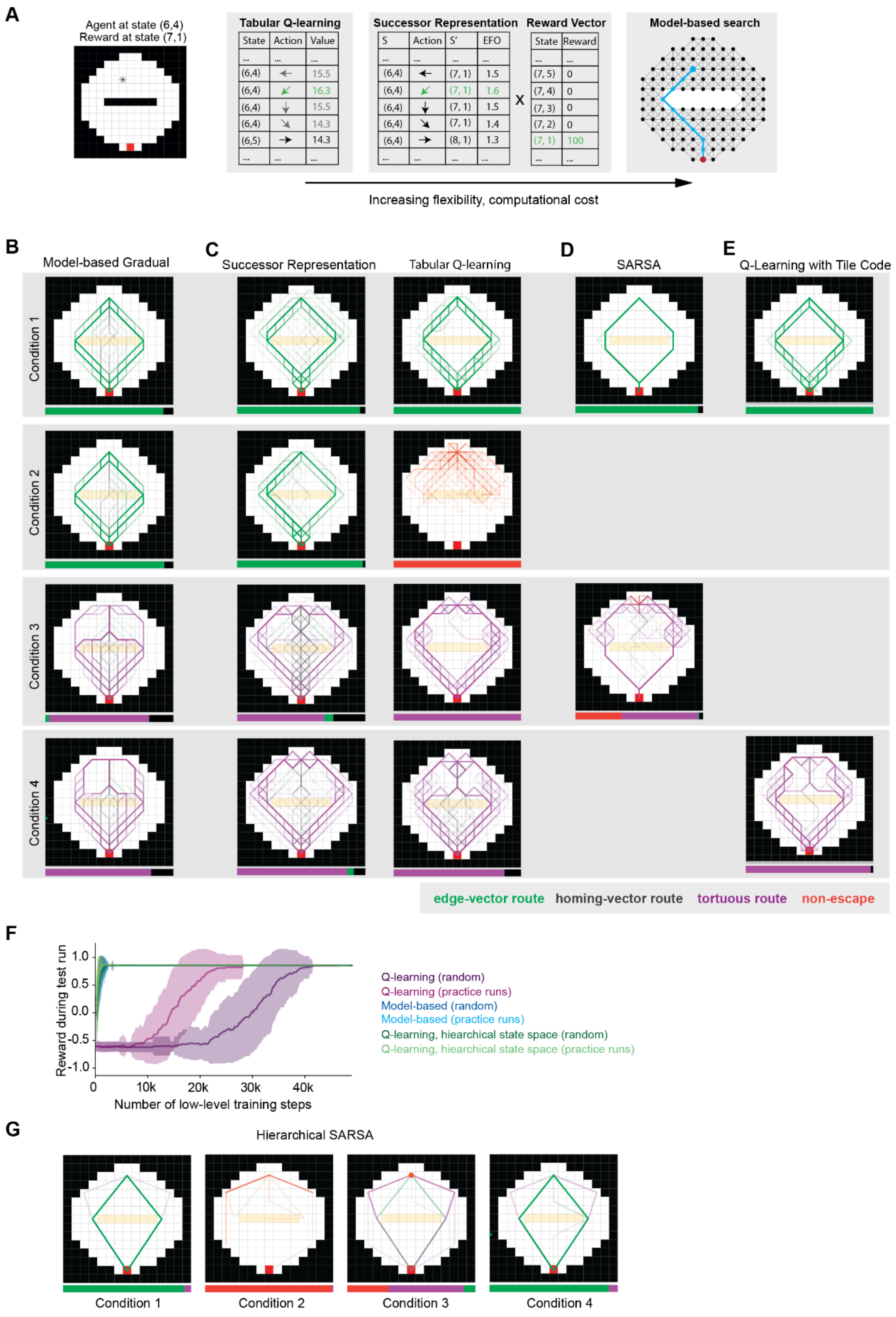

Figure S5: Reinforcement learning models of mouse escape behavior, Related to Figure 5

(A) Schematic of the core reinforcement learning models that we used. Q-learning updates a table of values for each state-action pair. Successor Representation updates a matrix recording how much each state predicts future occupancy in each other state, as well as a table of how much reward is in each state. Our model-based agents update a list of states, a graph of connections between each state, and a label of the amount of reward in each state. **(B)** Results for the gradual-

learning model-based agent. This agent uses the past 15 observations of each edge in the graph to decide if two adjacent states are connected or blocked by a barrier. Escape runs from all three trials of all 100 random seeds in all four conditions. All trials are superimposed, with high transparency. Bars under each plot show the proportion of each type of escape. **(C)** Results for the successor representation and Q learning, with random exploration (no practice runs). **(D)** Results for the SARSA agent. This agent is similar to tabular Q-learning, but it learns *on-policy*, meaning that the policy it converges to depends on its exploration policy. **(E)** Results for Q-learning with tile coding. Instead of a representing each state individually, tile-coding agent represents its approximate location with a set of n x n tiles (Methods). We used the coarsest-grained features with which the agent was still able to find a route to shelter was used (both 2x2 and 3x3 tiles, at once). **(F)** Learning curves plot the amount of reward received from a trial run in the test map (going from threat zone to shelter) over number of training steps. For the learning curves, there is no pre-test phase; the agent is simply dropped in the threat zone. The trial terminates when the agent reaches the shelter or at a maximum number of 50 steps. Since negative reward is given at each non-reward state (Methods), the minimum reward is approximately -0.5. **(G)** Results with the hierarchical state-space agent using the SARSA algorithm, with practice runs during exploration. Assuming a homing-vector agent takes over when this agent fails to come up with a route, this agent qualitatively matches mouse behavior on all four conditions: edge vectors in condition 1 and 4 and failure to learn a route in conditions 2 and 3. The orange dot in condition 3 indicates selecting an invalid action.

Supplemental References

S1 - Paxinos, G., & Franklin, K. B. J. (2019). *Paxinos and franklin's the mouse brain in stereotaxic coordinates*. Academic Press.

S2 - Allen Institute for Brain Science. (2015). Allen mouse brain atlas. https://mouse.brain-map.org/static/ atlas

S3 - Shamash, P., Carandini, M., Harris, K., & Steinmetz, N. (2018). A tool for analyzing electrode tracks from slice histology. *bioRxiv*.

S4 - Burnham, K. P., & Anderson, D. R. (2004). Multimodel inference. Sociol. Methods Res., 33(2), 261–304.
