# Supplementary material for: Mice identify subgoal locations through an action-driven mapping process
Source: Neuron. 2023 Jun 21;111(12):1966–1978.e8. doi: 10.1016/j.neuron.2023.03.034 (PMC10636595; doi:10.1016/j.neuron.2023.03.034)
Supplement: Document 2. Article plus supplemental information [file mmc8.pdf]

Neuron

Article

Mice identify subgoal locations through an action-driven mapping process

Highlights

- Interruption of obstacle-directed runs during exploration prevents subgoal learning
- Subgoal selection during escape depends on the mouse's position in the environment
- A dual-system reinforcement learning agent replicates the mouse behavior

Authors

Philip Shamash, Sebastian Lee, Andrew M. Saxe, Tiago Branco

Correspondence

t.branco@ucl.ac.uk

In brief

Shamash et al. show that mice use an action-driven process to learn useful subgoals and integrate them into a cognitive map of the environment that is used to escape to shelter.

Shamash et al., 2023, Neuron *111*, 1966–1978
June 21, 2023 © 2023 The Author(s). Published by Elsevier Inc.
https://doi.org/10.1016/j.neuron.2023.03.034

CellPress

CellPress
OPEN ACCESS

Neuron

Article

Mice identify subgoal locations through an action-driven mapping process

Philip Shamash,[1] Sebastian Lee,[2] Andrew M. Saxe,[2] and Tiago Branco[1,3,*]
[1]UCL Sainsbury Wellcome Centre for Neural Circuits and Behaviour, London W1T 4JG, UK
[2]UCL Gatsby Computational Neuroscience Unit, London W1T 4JG, UK
[3]Lead contact
*Correspondence: t.branco@ucl.ac.uk
https://doi.org/10.1016/j.neuron.2023.03.034

SUMMARY

Mammals form mental maps of the environments by exploring their surroundings. Here, we investigate which elements of exploration are important for this process. We studied mouse escape behavior, in which mice are known to memorize subgoal locations—obstacle edges—to execute efficient escape routes to shelter. To test the role of exploratory actions, we developed closed-loop neural-stimulation protocols for interrupting various actions while mice explored. We found that blocking running movements directed at obstacle edges prevented subgoal learning; however, blocking several control movements had no effect. Reinforcement learning simulations and analysis of spatial data show that artificial agents can match these results if they have a region-level spatial representation and explore with object-directed movements. We conclude that mice employ an action-driven process for integrating subgoals into a hierarchical cognitive map. These findings broaden our understanding of the cognitive toolkit that mammals use to acquire spatial knowledge.

INTRODUCTION

A fundamental ability of mobile animals is to learn the location of resources and how to get there. This can, in principle, be done using a variety of strategies. At one end, the behaviorist framework focuses on the importance of repeating actions. Mazes can be solved by learning the correct movements directly in a "stimulus-response sequence."[1,2] At the opposite end, the cognitive map theory proposes that animals have mental maps of their environments that they can query to navigate to goals.[3] In this framework, a spatial map is learned through an innate capacity to map observations and is used to derive novel actions.[4] These two strategies are thought to be separate processes in the brain, with the striatum responsible for repeating successful movements and targeting landmarks and the hippocampus for constructing an internal map of the environment.[5,6]

Cognitive maps are powerful because they decouple actions from spatial learning, allowing the computation of routes in an allocentric (spatial-location-centered) reference frame. Models of this class generally ignore the motivation underlying the learner's exploration and use "random agents" that select movements from a distribution of cardinal directions to map the environment.[7–10] Similarly, paradigmatic experiments in this vein focus on the cues rather than the actions that animals use to pinpoint locations and rely on sessions that end when the animal finds the reward.[2,11–13] This contrasts starkly with the way animals explore natural environments. Mice, for example, move in a highly structured manner, punctuating investigatory bouts along boundaries with rapid lunges to familiar, enclosed spaces or visually salient objects.[14] It thus seems plausible that the sensorimotor tendencies of each species play an important role in identifying important locations or compartments within the map rather than serving a fully independent function.[15–18]

The homing behavior of rodents offers a powerful window into the relationship between spontaneous exploration patterns and spatial cognition.[19] Within minutes of entering a new environment, rodents rapidly identify and memorize sheltering locations,[20] spontaneously shuttle back and forth between the outside and the "home,"[2–22] and respond to threatening stimuli by running directly to shelter.[23] Homing behavior is also sophisticated enough to involve map-based computations of multi-step escape routes. Shamash et al.[22] recently showed that mice learn to escape past obstacles by memorizing allocentric subgoal locations at the obstacle edges and that this learning was correlated with the execution of a particular sensorimotor action during exploration—spontaneous running movements targeting the obstacle edge. This raises the hypothesis that the execution of specific exploratory actions is important for learning elements of a cognitive map.

Here, we directly test this hypothesis by investigating whether spontaneous edge-directed runs are necessary for subgoal learning. We use closed-loop neural manipulations to precisely interrupt these runs during exploration and then examine the effect on the use of subgoals during escape behavior. We demonstrate that subgoal learning is action driven in nature and that it relies on a mapping capacity. We then use reinforcement learning (RL) models to identify the computational principles underlying this learning process. Overall, we suggest that

 © 2023 The Author(s). Published by Elsevier Inc.
This is an open access article under the CC BY license (http://creativecommons.org/licenses/by/4.0/).

spatial learning through natural exploration relies on a learning mechanism that combines both action- and map-based strategies.

RESULTS

Closed-loop optogenetic activation of premotor cortex to block spontaneous edge-vector runs

When mice are placed in an arena with a shelter and an obstacle, they spontaneously execute runs targeting the obstacle edge.[22] Our main aim here was to test the causal necessity of these runs in learning that the obstacle edge is a subgoal, i.e., a location that should be targeted to run past the obstacle to get to the shelter. We therefore designed a manipulation to prevent mice from executing spontaneous runs to an obstacle edge. To prevent confounding effects, our manipulation should not change the external environment, reduce the animal's opportunities to observe the environment, or create a place aversion. We found that closed-loop stimulation of premotor cortex (M2) fit all criteria. We expressed channelrhodopsin in excitatory neurons in the right M2 and performed optogenetic stimulation via an implanted optic fiber (Figures 1B and S1A). As previously reported,[24,25] stimulating M2 with a 2-s, 20-Hz pulse wave caused low-latency (<200 ms) deceleration, halting, and leftward turning motion (Figure S1B; Video S1). This stimulation protocol did not generate place aversion in a two-chamber place-preference assay (Figure S1D). We thus leveraged this approach to specifically interrupt edge-vector runs during spontaneous exploration. Using online video tracking, we set up a virtual "trip wire" between the threat area and the left obstacle edge; whenever mice crossed this line while moving in the direction of the edge, a 2-s light pulse was automatically delivered (Figures 1C and S1C; Video S1). All other movements, including runs to the left edge along the obstacle or from the shelter, were not interrupted by laser stimulation.

We divided injected and implanted animals into a laser-on and a control, laser-off group. Both groups explored a circular platform with a shelter and an obstacle for 20 min (n = 8 mice/sessions; Figures S2A and S2B). During this time, all mice located the shelter and visited the entire platform, including the obstacle (Figures S3F and S3H). In agreement with previous results,[22] all mice in the laser-off group executed continuous running movements from the threat area toward the shelter area ("homing runs"; No. per session: 6 [5, 8.25], median [IQR]; Figures 1A, S3G, and S3I). These included at least one homing run that directly targeted an obstacle edge ("edge-vector runs"; No. per session: 1.5 [1, 2.25], median [IQR]); Figures 1A and S3J; Video S1). Mice in the laser-on group triggered 3.5 [2.75, 6] (median [IQR]) laser stimulation trials, lasting 20 [16, 26] s in total and interrupting all potential edge-vector runs (Figures 1D, S3G, and S3J). While mice in the laser-off group executed nearly direct paths between the threat area and the left obstacle edge, the paths taken by mice in the stimulation group were twice as long, reflecting the inaccessibility of edge-vector runs (Figure 1E). Exploratory behavior, however, was not reduced. Mice in the stimulation condition explored the obstacle, the edge, the threat area, and the entire arena as much as the control group (Figures 1F, S3F, and S3H).

Interrupting spontaneous edge-vector runs abolishes subgoal learning

We next measured the impact of blocking edge-vector runs on subgoal learning. After the 20 min exploration period, we elicited escape behavior using a threatening sound. Mice triggered the threat stimulus automatically by entering the threat zone and staying there for 1.5 s. Escape routes were quantified using a target score and classified as targeting the obstacle edge (edge vector) or the shelter (homing vector) (Figure 2B; see STAR Methods). First, we acquired a negative-control distribution by presenting threats to mice that explored an open-field environment with no obstacle (n = 8 mice; same viral injection and implantation procedure as above). As expected from previous work,[20] mice escaped by turning and running directly along the homing vector (Figures 2A and S2C; Video S2). Second, we examined escapes in a positive-control condition known to generate subgoal learning. After the laser-off group explored the arena with the obstacle and shelter for 20 min, we removed the obstacle and triggered escapes (2–30 min later, IQR: 8–17 min). We found that 42% of escapes were directed toward the obstacle edge location despite the obstacle being gone (edge vectors; 26 total escapes on the left side; more edge vectors than in the open field: $p = 0.003$, permutation test; Figures 2A and 2C; right-side escapes shown in Figure S2D; Video S3). This result is consistent with Shamash et al.,[22] which found that these edge-vector escapes reflect the memorization of a subgoal location. Third, we tested the laser-on group, which explored with an obstacle and shelter but had their exploratory edge-vector runs interrupted. After removing the obstacle, threat-evoked escape routes were similar to the paths taken in the open-field condition rather than the subgoal-learning group (13% edge vectors; 23 escapes [left side]; fewer edge vectors than in the laser-off condition: $p = 0.03$, and not significantly more edge vectors than in the open field: $p = 0.2$, permutation tests; Figures 2A and 2C; Video S3). Thus, interrupting spontaneous edge-vector runs abolished subgoal learning.

An alternative explanation could be that mice did learn subgoals, but the stimulation during edge-vector runs taught them to avoid expressing edge-vector escapes. To address this, we repeated the stimulation experiment (n = 8 mice) but allowed mice to perform two spontaneous trip-wire crossings before subjecting them to the same edge-vector-blocking protocol as above (3 [1.75, 4.25] laser trials per session, median [IQR], lasting 16 [5.5, 26.5] s in total; Figures S3A–S3E; Video S4). Removing the obstacle and triggering escapes now revealed robust subgoal behavior (65% edge vectors; n = 23 escapes [left side]; more edge vectors than in the open field: $p = 3 \times 10^{-4}$, and not significantly fewer edge vectors than the laser-off condition: $p = 0.9$, permutation tests). This shows that our manipulation does not reduce the use of subgoals once they are learned and suggests that edge-vector runs are causally required for learning subgoals.

Blocking edge-to-shelter runs does not reduce subgoal learning

Spontaneous edge-vector runs were often followed by an edge-to-shelter run. After completing an edge-vector run, mice in the laser-off condition reached the shelter within 2.5 [1.7,10] s (median

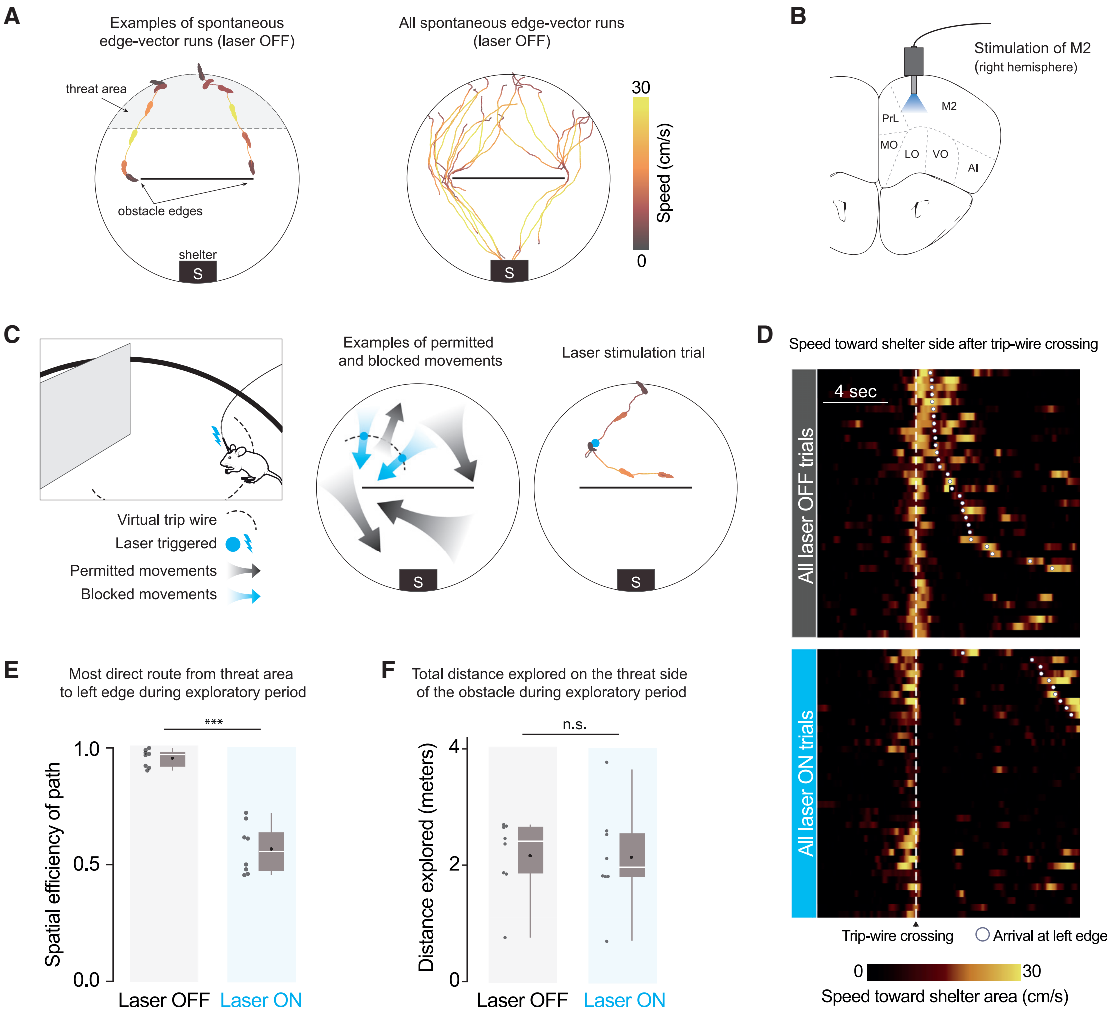

Figure 1. Closed-loop optogenetic activation of M2 interrupts spontaneous edge-vector runs

(A) Spontaneous edge-vector runs during the initial exploration period (continuous turn-and-run movements, starting in the threat area and stopping at or moving past the obstacle edge); n = 8 mice.

(B) Schematic illustrating optic fiber placement in the right premotor cortex. M2, supplementary motor cortex (premotor cortex); PrL, prelimbic cortex; MO/LO/VO, medial/lateral/ventral orbital cortex; AI, agranular insular cortex.

(C) On crossing a virtual trip wire (dashed line) during exploration, mice automatically received a 2-s, 20-Hz light pulse. This caused a stopping and leftward-turning motion preventing the mice from reaching the obstacle edge. In the example trial, the mouse ran to the right side of the platform after the stimulation. Mouse drawing: scidraw.io.

(D) All trip-wire crossings, with and without laser stimulation, ordered by time of arrival to the left obstacle edge. Note that mice must be moving toward the shelter area (i.e., southward) to trigger the trip wire.

(E) Spatial efficiency is the ratio of the straight-line path to the length of the path taken. White horizontal lines, median; black dots, mean; gray boxes, first and third quartiles; gray vertical lines, range. Each dot represents one mouse/session. $p = 5 \times 10^{-5}$, one-tailed permutation test.

(F) Distance explored on the threat half: p = 0.5, one-tailed permutation test; n = 8 mice in each group.

[IQR]), generally taking direct paths (spatial efficiency: 0.87 [0.47, 0.95]; 1.0 corresponds to the direct path; Figures S3F and S3H). We therefore considered whether edge-vector runs support subgoal learning because they are part of a sequence of actions that quickly brings the mouse from the threat zone to the shelter. To test this, we modified the stimulation experiment to block the second phase of the threat-area-to-edge-to-shelter sequence by placing the trip wire in a location that stopped movements from the left obstacle edge toward the shelter (n = 8 mice; 3 [2, 3.25] laser trials per session, median [IQR], lasting 25 [20, 30] s in total; Figures 3A and S3D–S3I; Video S4). This manipulation resulted in edge-vector runs on the left side being followed by long, slow paths to shelter (seconds to shelter: 29 [18, 55]; spatial efficiency: 0.28 [0.13, 0.37]; slower than the laser-off condition: $p = 1 \times 10^{-3}$; less spatially efficient than the laser-off condition: $p = 2 \times 10^{-3}$, permutation tests; Figures S3F and S3H). Despite this effect, removing the obstacle and triggering escapes revealed robust subgoal behavior (55% edge vectors; n = 23 escapes [left side]; Figures 3B and 3C; more edge vectors than in the open field: $p = 1 \times 10^{-4}$, and not significantly fewer edge vectors than the

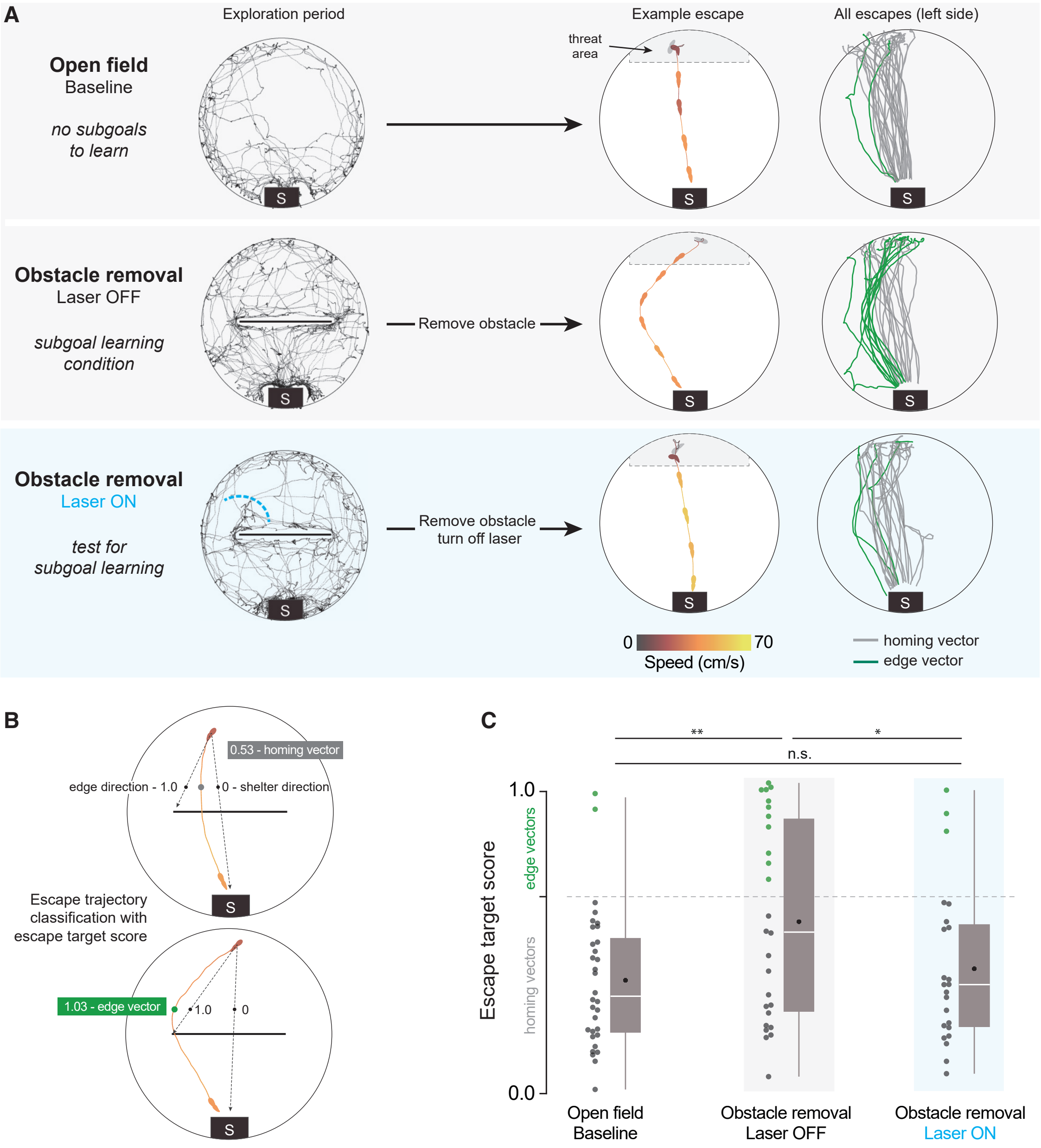

Figure 2. Interrupting spontaneous edge-vector runs abolishes subgoal learning

(A) Black traces show exploration during an example session (open field: 10 min, obstacle removal: 20 min). Lines and silhouette traces show escape routes from threat onset to shelter arrival; open field: 29 escapes; obstacle removal (laser off): 26 escapes; obstacle removal (laser on): 23 escapes. All: n = 8 mice.

(B) The initial escape target is the vector from escape initiation to 10 cm in front of the obstacle (black dots), normalized between 0 (shelter direction) and 1 (obstacle edge direction).

(C) Escape target scores over 0.65 are classified as edge vectors; scores under 0.65 are classified as homing vectors (as in Shamash et al.[22]). Obstacle removal (laser off) vs. open field: $p = 0.003$; obstacle removal (laser on) vs. open field: $p = 0.2$; Obstacle removal (laser off) vs. obstacle removal (laser on): $p = 0.03$, one-tailed permutation tests on proportion of edge-vector escapes.

laser-off condition: $p = 0.8$, permutation tests). Thus, for their causal role in subgoal learning, edge-vector runs do not need to be rapidly followed by the extrinsic reward of entering the shelter. This result also supports the argument that optogenetic stimulation at the left edge does not teach the mice to avoid passing by that location during escapes.

Subgoal-escape start points are determined by spatial rules

The results from the previous experiment suggest that learning subgoals with edge-vector runs is not simply a matter of reinforcing actions that lead to the shelter. This fits with the finding by Shamash et al.[22] that subgoals in this context are stored as

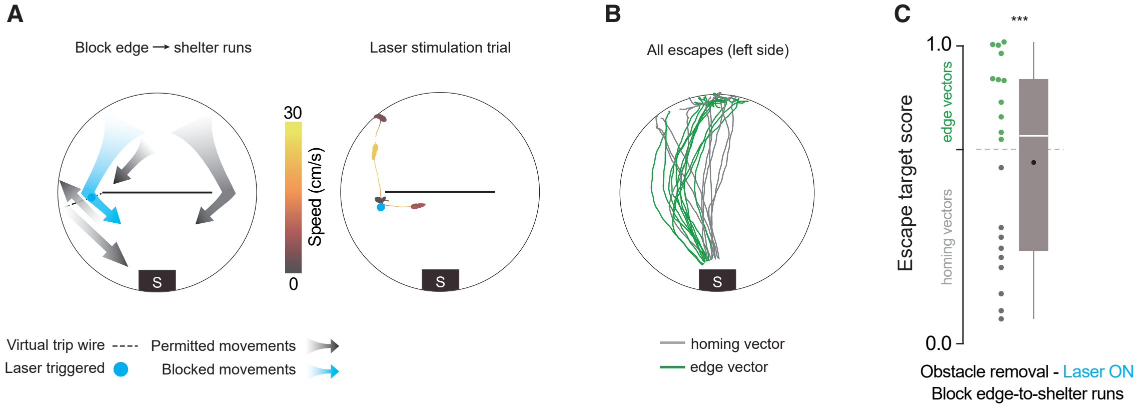

Figure 3. Blocking edge-to-shelter runs does not diminish subgoal learning

(A) Blocking left-edge-to-shelter runs by activating M2 at the obstacle edge. In the example trial, the mouse was stimulated for 10 s and then ran toward the center of the platform.

(B) Escapes after obstacle removal. n = 8 mice, 23 escapes (left side).

(C) Obstacle removal (block edge-to-shelter) vs. open field: $p = 1 \times 10^{-4}$; vs. obstacle removal (block edge vectors): p = 0.03; vs. obstacle removal (laser off): p = 0.8; one-tailed permutation tests on proportion of edge-vector escapes.

allocentric locations rather than egocentric movements and raises the possibility that the learning process combines actions and spatial information. To explore this further, we investigated the rules governing the set of locations from which mice initiate memory-guided subgoal escapes—the "initiation set" of subgoal escapes. We aimed to determine whether the initiation set was (1) spread indiscriminately throughout the environment, (2) restricted to the vicinity of previous edge-vector-run start positions, or (3) related to the spatial layout of the environment independent of past actions. Option 1 would be expected if mice learned to execute edge-vector actions without taking into account their starting location, option 2 if mice learned to repeat edge-vector actions based on proximity to previous successful actions, and option 3 if mice selected the subgoal strategy through a map-based process. We first repeated the obstacle removal experiment but now elicited escapes from in front of the obstacle location, near the shelter (n = 8 mice with no laser stimulation, 28 escapes; Figure S4A). From this starting point, mice did not escape by running toward a subgoal location but instead fled directly to shelter, suggesting that the initiation set is spatially confined rather than indiscriminate.

Next, we tested whether the initiation set is confined to the area in which spontaneous edge-vector homing runs have previously occurred. We modified our laser stimulation experiment with a new trip-wire location, so that edge-vector runs were allowed from a section of the arena next to the threat zone but were interrupted if they started within the threat zone (n = 8 mice; 2 [1.75, 4] laser trials per session, median [IQR], lasting 4 [6, 9] s; Figures 4A, 4B, and Figures S3D–S3I; Video S4). As before, laser stimulation succeeded in blocking edge-vector runs from the threat zone (Figure S3I). In this configuration, however, mice were still able to execute edge-vector runs starting from the area to the left of the threat zone (leftmost gray arrow in Figure 4A; Figure S6D). Removing the obstacle and triggering escapes in this cohort revealed robust subgoal behavior (63% edge vectors; n = 19 escapes [left side]; Figures 4B and 4C; more edge vectors than in the open field: $p = 6 \times 10^{-4}$, and not significantly fewer edge vectors than the laser-off condition: p = 0.8, permutation tests). Thus, the initiation set for subgoal escapes extends beyond the locations in which successful edge-vector runs have been initiated (Figure 4B, inset). This result also reaffirms that optogenetic stimulation does not teach mice to avoid paths that are blocked by laser stimulation during exploration.

To more precisely determine the impact of spatial location on subgoal behavior, we ran the obstacle removal experiment with a larger threat zone, located between the obstacle location and the original threat zone (n = 8 mice, 53 escapes; no laser stimulation; Figures 4D and S4D). We then combined these escapes with the original threat zone data and used logistic regression to test the relationship between the location of escape onset and subgoal use (n = 40 total sessions, 207 escapes; Figures 4E–4H). We found that being closer to previous edge-vector runs was not significantly related to the likelihood of executing edge-vector escapes (McFadden's pseudo-$R2$ = 0.086; p = 0.5, permutation test; Figures 4G, S4C, and S4D); on the contrary, this non-significant relationship tended toward greater distance from an edge-vector run predicting a higher likelihood of edge-vector escapes. In contrast, several spatial metrics were effective predictors of edge-vector escape probability (Figures 4F, 4G, and S4C–S4E). These include the distance from the obstacle, distance from the central axis of the platform (the axis perpendicular to the obstacle), distance from the shelter, and angle between the edge-vector and homing-vector paths. Thus, the initiation set is defined in relation to the layout of the environment rather than proximity to previous successful actions.

We next analyzed whether a two-dimensional (2D) spatial-location predictor fit the data better than a one-dimensional (1D) predictor by applying Akaike Information Criterion (AIC) analysis to the logistic regression model (Figure 4H). If the initiation set were fully explained by the mouse's perception of which

CellPress
OPEN ACCESS

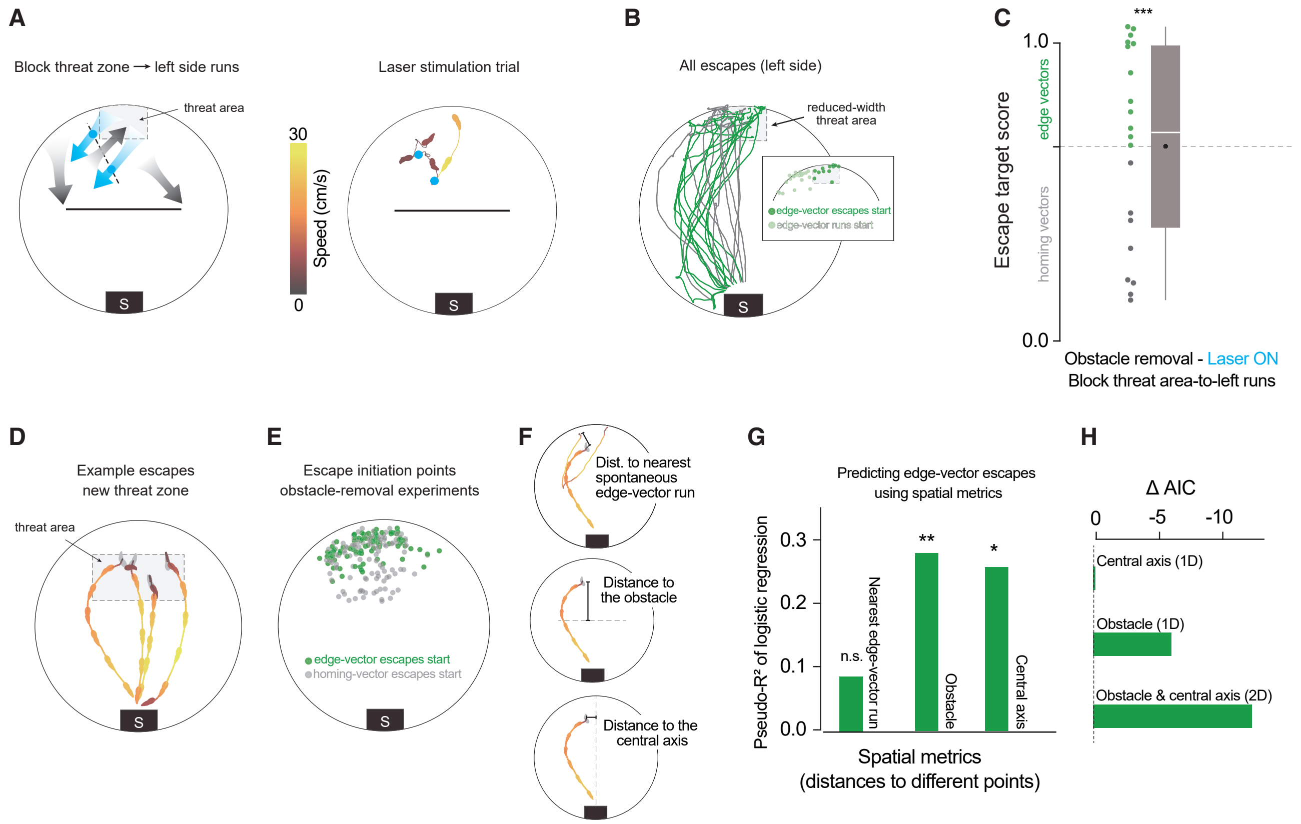

Figure 4. Subgoal-escape start points are determined by spatial rules

(A) Blocking threat-zone-to-left-side runs by changing the trip-wire location and width of the threat zone. In the example trial, there were two consecutive trip-wire crossings (2-s stimulations), after which the mouse moved back toward the threat zone.

(B) Escapes after obstacle removal. The reduced-width threat zone ensured that mice would need to cross the deactivated trip wire in order to execute edge-vector escapes; n = 8 mice, 19 escapes (left side). Inset: all start locations for spontaneous edge-vector runs (light green) and subsequent edge-vector escapes (dark green).

(C) Obstacle removal (block threat-zone-to-left-side) vs. open field: $p = 6 \times 10^{-4}$; vs. obstacle removal (block edge vectors): p = 0.01; vs. obstacle removal (laser off): p = 0.8, one-tailed permutation tests on proportion of edge-vector escapes.

(D) Four example escapes triggered after obstacle removal with the threat zone in a new position.

(E) Pooled data from all obstacle-removal experiments (excepted the block-edge-vectors experiment). Escapes on both the left and right sides are shown. Right-sided escapes are flipped horizontally for visualization, and thus, all the green dots can be seen as left-edge vectors. Each dot represents one escape; n = 40 sessions, 207 escapes.

(F) Illustration of three spatial metrics used to predict the likelihood of executing an edge-vector escape. Silhouettes in each arena image are an example escape; orange trajectories in the top image illustrate the corresponding history of edge-vector runs in the exploration period. Black bar shows the distance being measured.

(G) McFadden's pseudo-R^2 measures the strength of the relationship between each metric and the odds of executing edge-vector escapes. Values of 0.2–0.4 represent "excellent fit."[26] Distances are measured from the escape initiation point of each escape. For the distance to the nearest spontaneous edge-vector run start point, only runs toward the same side as the escape are considered. Distance to the nearest start point of a spontaneous edge-vector run: pseudo-R^2 = 0.086; p = 0.5. Distance to the obstacle: pseudo-R^2 = 0.28; p = 0.007. Distance to the central axis: pseudo-R^2 = 0.26; p = 0.01.

(H) Akaike Information Criterion (AIC) analysis on a logistic regression with different predictors. Decreases in AIC represent better model fit and include a penalty for using additional predictors; ΔAICi = AICi – AICmin, where AICmin here is the AIC from the model with the single distance-from-central-axis predictor.

side of the obstacle it is on or of how close to the shelter it is, then adding additional spatial predictors should not improve the model (i.e., AIC should increase). On the other hand, if mice use their 2D position within the environment to select whether to use a subgoal, then using 2D spatial information should improve the model (i.e., AIC should decrease). In line with this possibility, using only distance from the obstacle (i.e., distance along the y axis) or distance from the central axis (i.e., distance along the x axis) produced AIC scores of 206.8 and 212.9, respectively, whereas the AIC for using both dimensions as input was 200.5. The magnitude of the AIC decrease (6.3) indicates that the combined 2D model has considerably more support than either 1D model.[27] We found similar results with distance from the shelter plus distance to the central axis as a predictor (Figure S4F; see Figure S4E for an alternative analysis). These analyses further support the hypothesis that the selection between subgoal routes and direct routes is modulated by their 2D starting position within the arena.

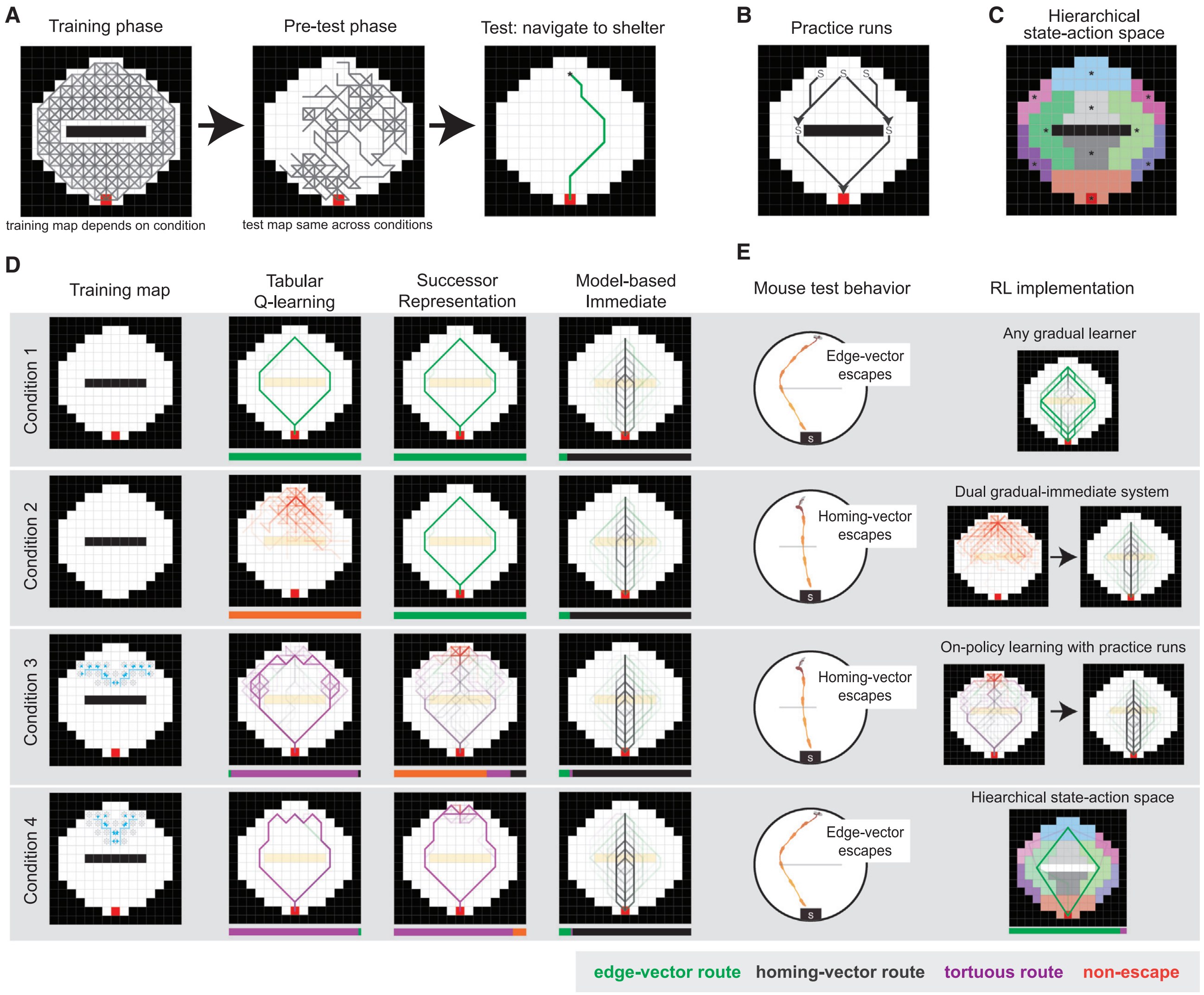

Figure 5. Reinforcement learning models of mouse escape behavior

(A) Schematic illustrating the training, pre-test, and testing phases. Gray traces represent paths taken during exploration by the RL agents (training map shown is the map used in condition 1). Accessible states are white, blocked states are black, and accessible rewarded states are red. In the training phase, agents have sufficient exploration for all 100 random seeds to learn a path from the threat zone to the shelter. Middle: a representative exploration trace from the pre-test phase. Right: an example "escape" trajectory from the threat zone (asterisk) to the shelter (red square).

(B) Illustration of the practice runs included in the training phase. Each "S" represents a start point for the hard-coded action sequence, and each arrowhead shows the terminal state. The sequences were triggered with probability p = 0.2 upon entering each start state.

(C) Segmented arena used for the hierarchical state-space agent. Each colored region represents a distinct state. After selecting a neighboring high-level region to move to, the agent moves from its current location to the region central location indicated by the asterisks.

(D) Escape runs from all seeds in all four conditions for the Q-learning, Successor Representation, and model-based (immediate learner) agents. All trials are superimposed. Bar chart below each plot shows the proportion of each type of escape. Edge-vector routes go directly to the obstacle edge; homing-vector routes go directly toward the shelter; tortuous routes go around both the obstacle and the trip wire; non-escapes do not arrive at the shelter. In the training map of conditions 3 and 4, the one-way trip wire is represented by the blue line, and the blue arrows indicate the blocked transitions.

(E) Qualitative mouse behavior for each condition (left) and illustration of the type of RL agent that matches this behavior (right). Condition 1: gradual model-based shown; condition 2: Q-learning and immediate model-based shown; condition 3: SR and immediate model-based shown; condition 4: hierarchical-state-space Q-learning shown.

A dual RL system matches mouse behavior after obstacle removal

We next aimed to determine the computational principles behind subgoal learning by identifying RL modeling strategies that can qualitatively capture the behavior. We used a spectrum of RL algorithms previously used to model navigation[28,29] in a tractable grid-world environment based on our experimental setup (Figure 5A; STAR Methods). The three core algorithms we used were model-free tabular Q-learning, the Successor Representation (SR),[30] and model-based tree search (see Figure S5A and

Table 1. Number of training steps needed to learn escape routes

Algorithm	Exploration	No. steps to learn escape routes
Tabular Q-learning	random	45k
Tabular Q-learning	random + practice runs	30k
Hierarchical Q-learning	random	2.5k
Hierarchical Q-learning	random + practice runs	1.5k
Tile-coding Q-learning	random	285k
Successor Representation	random	125k
Successor Representation	random + practice runs	20k
Model-based (immediate)	random + practice runs	3k
Model-based (gradual)	random + practice runs	3k
Tabular SARSA	random + practice runs	35k
Hierarchical SARSA	random + practice runs	2k

STAR Methods for detailed descriptions). The tabular Q-learning agent incrementally learns the value of each of the 944 state-action pairs (e.g., "go northwest from the shelter state") based on its history of receiving rewards. The SR also computes state-action values but updates two separate representations: a spatial representation measuring which locations follow each state-action pair and a reward representation. It then combines this information to compute the estimated value of each state-action pair. Third, the model-based agent does not update action values but instead updates a graphical representation of the arena and searches through this graph to calculate optimal routes to the reward. This model is different from the other two algorithms in two main ways: it uses model-based search, and it updates the model immediately after visiting a state. To facilitate determining the role of these two properties when performing model comparisons, we also included a model-based agent that updates its model gradually, using the past 15 observations of each graph edge to decide whether two adjacent states are connected or blocked by a barrier.

Similar to the experiments in mice, all simulations included a training map (e.g., the arena with an obstacle present) and a test map (e.g., the arena with the obstacle removed) and took place over three phases (Figure 5A). In the "training phase," the agent explored a training map for a duration long enough to learn a route from the threat zone to the shelter (Table 1). Importantly, this phase also included stochastically generated practice-run sequences from the threat zone to the obstacle edge and from here to the shelter to mimic the natural exploratory pattern observed in mice (Figure 5B). This was followed by the "pre-test phase," which took place in the test map. In this phase, the agent started in the shelter and executed a random-exploration movement policy until reaching the threat zone. Finally, there was a "test phase" executing the learned policy in the test map, starting from the threat zone. We selected four particularly revealing behavioral and optogenetic experiments to model *in silico* using this procedure. All test maps hade a shelter and no obstacle, and, therefore, the only difference between the four experimental conditions was the training map.

The first condition was the basic obstacle removal experiment (Figures 2, 5D, and 5E), where the training map had an obstacle and a shelter (i.e., a reward). Similar to mice, the Q-learning, SR, and gradual model-based (MB-G; Figure S5B) agents all exhibited persistent escape routes around the obstacle in the test phase. The immediate-learning model-based (MB-I), on the other hand, was able to update its model during the test-map exploration and compute the new, fastest route to the shelter 94% of the time. The differentiating factor here was whether the agents updated their policy immediately (MB-I) versus incrementally or stochastically (all others). In the latter case, the pre-test exploration was too brief to learn the homing-vector path.

In the second condition, the training map had an obstacle but no shelter (Figure 5D). Mice in this experiment[22] failed to learn edge-vector routes and instead escaped using homing vectors. The only agent to take homing vectors here was the agent that did not execute edge vectors in condition 1 (MB-I, 92% homing vectors). The remaining agents differed in their behavior. The SR and MB-G agents learned edge vectors because of their ability to separately solve spatial-learning problems even in the absence of reward (SR, 100% edge vectors, Figure 5D; 93% edge vectors, Figure S5B). The Q-learning agent failed to learn edge vectors or any alternative escape route because it cannot learn without reward in the environment (100% non-escape; Figure 5D).

Overall, mice exhibited a pattern unlike any of these RL agents. Mice failed to immediately learn a homing-vector path in condition 1, but they did immediately learn the homing-vector path when they did not have a memorized policy in place (condition 2). For the RL models, this represents a paradox: the models that learned fast enough to run straight to shelter in condition 2 would also do so in condition 1. One solution to this paradox is a dual system that can switch between flexible and inflexible learners depending on the situation.[31,32] We implemented this solution in an agent containing both a Q-learning and an MB-I system. When the Q-learning model suggested an action with a positive value above a threshold, the agent would take that action. If no such action was available, as in condition 2, the MB-I system was invoked to find a novel route. This agent was able to match mouse behavior on conditions 1 and 2 (Figure 5E).

Behavior with the full trip wire is matched with non-uniform exploration and on-policy learning

Next, we added the optogenetic trip wire to our modeling environment. In addition to the obstacle and shelter, the training map now contained paths from the threat area to the obstacle edge that were blocked one way. Mice in this experiment again failed to learn edge-vector routes (Figure 2). We were thus looking for a gradual learning system that failed to learn viable escapes with the trip wire present, thereby triggering the backup immediate learner. For Q-learning and MB-G, the trip wire simply added an additional detour. These agents learned tortuous routes around both the trip wire and the obstacle (Q-learning: 98% tortuous routes around both trip wire and obstacle, Figure 5D; MB-G: 78% tortuous routes; 19% homing vectors;

Figure S8B), indicating that models that memorized routes around the physical barrier would tend to do the same with the trip wire.

In contrast, the SR agent did not have this problem; it learned routes around the obstacle in condition 1 but failed to learn with the trip wire (70% non-escape; Figure 5D). This happened for two reasons. The first reason was the practice runs in the training phase. With a fully random policy, the SR agent learned routes in condition 3 just as quickly as routes in condition 1 (Figure S5C). Thus, it was the practice edge-vector runs that predisposed this agent to learn edge-vector routes faster than other, arbitrary paths through space. The second reason was that, unlike Q-learning, our SR implementation was an on-policy learner.[33] This means that value that it attributed to an action depended on how often that action led to the shelter during the training period. Because uninterrupted practice-run action sequences were possible only in condition 1, edge-vector actions accumulated high values faster than the meandering actions leading around the trip wire in condition 3. In line with this explanation, we found that an on-policy variant of Q-learning (SARSA) with practice runs behaved similarly to the SR: it also often failed to find routes in condition 3, but not condition 1 (Figure S5D). Thus, the pattern of exploration that we observed in mice—slow meandering exploration punctuated by rapid edge- and shelter-directed runs—could explain why an on-policy learner would learn edge-vector runs in condition 1 but fail to learn a route in condition 3.

Behavior with the partial trip wire is matched with state-action abstraction

Our final condition (condition 4) mimicked the optogenetics experiment in Figure 4. This partial trip wire blocked edge-vector runs from the threat zone itself, but not from other, nearby locations. In this condition, mice learned direct edge-vector escapes. The gradual-learning RL agents, on the other hand, all executed tortuous routes around both the trip wire and the obstacle (Q-learning: 98% tortuous routes; SR: 90% tortuous routes; MB-G: 81% tortuous routes, 17% homing vectors; Figures 5D and S5B). To match mouse behavior on both conditions 3 and 4, an RL agent would need to run through the line where the trip wire was during training instead of taking a step-by-step route around it. In addition, it would have to infer the availability of this direct edge-vector route based on nearby, but non-identical practice runs during the training phase.

We reasoned that an agent with a coarse-grained state space could possess these features. We first tried implementing Q-learning with a coarse-grained state representation designed to promote spatial generalization (tile coding).[34] This agent's behavior, however, was not substantively different from tabular Q-learning (98% tortuous routes; Figure S5E). Next, we tried a more targeted state-action abstraction protocol. We divided the state space into regions of grid squares (e.g., the shelter area, the left obstacle edge area) and the action space into vectors connecting those regions (Figure 5C) (note that we could have used a more sophisticated state-action abstraction scheme such as the options framework[35] but found this to be the most direct solution to condition 4). This Hierarchical State Space (HSS) Q-learning agent explored using the same random walk policy on the full-resolution training map but updated its controller only with respect to transitions between the high-level regions. We found that this agent was able to learn edge-vector escapes even with the partial trip wire in place (94% edge vectors; Figure 5E). Notably, the HSS agent could learn a valuable "threat area to obstacle edge area" action without ever having taken that action from the exact grid cell where the escape is triggered. These high-level actions also better matched the smooth, biphasic escape trajectories we saw in mice and generated a much faster learning profile (Figure S5F). In addition, the regional state representation fit well with our finding that mice use a spatially defined "subgoal initiation set" (see Figure 4).

To summarize, the vanilla RL agents we tested were not effective at matching mouse behavior across all experimental conditions. To achieve this, our simulations suggested that we need an agent that:

1. includes a gradual-learning system.
2. does not fully separate spatial and reward learning.
3. abstracts over regions of space and the actions connecting those regions.
4. has an immediate-learning system (e.g., hardcoded homing-vector policy, MB-I) in parallel with the gradual system that comes online when the gradual learner has no valuable action.
5. experiences non-uniform exploration, with rapid and direct practice runs toward the obstacle edges and shelter.

Having defined these five key computational principles, we then built an agent with all these properties. This agent included a gradual learning system that directly learned action values in an on-policy manner (i.e., the SARSA algorithm) within the high-level state-action space introduced above. The agent performed practice runs during exploration, and we assume that it switched to a default MB-I agent in conditions with high failure rates. We found that this agent could qualitatively match mouse behavior on all four conditions, executing persistent edge vectors in conditions 1 and 4 and frequently failing to escape in conditions 2 and 3 (Figure S5G).

DISCUSSION

When a mouse investigates a new environment, it does not act like a "random agent." Instead, its exploration consists of purposive, extended, sensorimotor actions. In this work, we have demonstrated that one such class of movements—running to an obstacle edge that grants direct access to a goal—plays a causal role in the process of gaining useful spatial information about the environment.

Our previous work has showed that during 20 min of exploration with a shelter and an obstacle, mice memorize subgoals at the obstacle edge location.[22] This is revealed by removing the obstacle and presenting threats, which causes mice to initiate escapes by running to the location of an edge that is no longer there. To explain this allocentric behavior, typical spatial learning models would rely on two steps: (1) constructing an internal map of space by observing how locations and obstructions in the

CellPress
OPEN ACCESS

environment are positioned relative to each other and (2) using this map to derive a useful subgoal location, computed either at decision time or in advance during rest.[36,37] This process is well suited for agents that learn by diffusing throughout their environment, be it randomly or with a bias toward unexplored territory.[38] However, it does not account for the prevalence of goal- and object-oriented actions in natural exploratory patterns.[14,39]

We thus explored a potential role for a third process: (3) executing "practice runs" to candidate subgoal locations during exploration. This idea follows from a strain of research in the cognitive sciences called sensorimotor enactivism,[40] which asserts that an explanation of learning should include not only how an animal extracts meaning from its sensory data but also how its actions are used to control this stream of data.[16–18,41,42] Here, we combined this principle—the importance of intrinsically motivated actions for learning—with the causal perturbation techniques and spatial behaviors available in rodent neuroscience. Specifically, we used closed-loop optogenetic stimulation of M2 to interrupt edge-vector practice runs and found that this manipulation abolished subgoal escape routes.

It is important to note that this effect does not inform us about the role that M2 may play in computing subgoals. This question is not the point of this study or the goal of manipulating M2 activity. Notably, three M2 stimulation protocols spared edge-vector runs, and these manipulations did not impair learning. Thus, stimulating M2 does not intrinsically affect spatial learning. Only when M2 stimulation interrupted practice edge-vector runs did we see the effect. Our results therefore indicate that the edge-vector actions themselves are necessary for triggering subgoal memorization. While the neural implementation remains unknown, our results open the door for future work elucidating the network of motor and spatial brain nuclei that implement subgoal memorization. The action driven mapping strategy we have uncovered suggests that the coordination between map and action-reinforcement systems might be tighter than previously thought, and thus, it will be particularly interesting to investigate interactions between the hippocampus and striatum during subgoal learning.

One interpretation of the need for practice runs in learning could be that subgoal behavior is a naturalistic form of operant conditioning. In this view, edge-vector runs are followed by reinforcement and then simply get repeated in response to threat. This framework could explain why edge-vector responses persist after obstacle removal: they are habits that have not yet been "extinguished." Moreover, the lack of effect of blocking edge-to-shelter runs fits with an instrumental chaining mechanism,[1,43] in which arrival at the obstacle edge itself acts as a reinforcer. However, subgoal learning diverges from instrumental learning in two ways: it operates within an allocentric framework (generally seen as distinct from an instrumental response strategy,[2,5,6,31] and it only requires 1–2 practice runs (even simple instrumental training takes tens of learning trials.[44] More importantly, the set of locations from which mice initiate subgoal escapes are defined by the mouse's spatial position relative to the obstacle and shelter and not by their proximity to previous edge-vector runs. The concepts of action and reinforcement are therefore insufficient for explaining subgoal memorization; an internal map of space must also be invoked.

There are several possible explanations of the initiation set's spatial arrangement, with subgoals executed when the mouse is farther back from the obstacle location and from the arena's central vertical axis. First, it could reflect the outcome of a spatial cost-benefit analysis: the preferred subgoal-escape starting points are in the locations where the subgoal route is almost as short as the homing vector. Second, it could indicate that the memory-guided escape strategy is only used when the animal is so far away from the shelter or obstacle's center that the animals know that they cannot rely on local visual cues. One final possibility is that the mouse clusters its spatial map of the arena into regions with similar features. In that case, subgoal actions might generalize across the back perimeter region but not to the region right in front of the obstacle.

To formalize the computational properties of subgoal learning, we performed RL modeling of four key behavioral and optogenetic experimental conditions. First, we found that models that update gradually—be it model-free or model-based—can match our persistent edge-vector escape result. Second, we found that mice exhibit differing levels of flexibility in different conditions and are thus best modeled through a dual-system agent. This dual agent included one system that updates a policy gradually and another that learns much more rapidly (at a greater computational cost). For the rapid learner, we used the MB-I system, though there is no principled reason why this needs to be a classical model-based system. One appealing alternative is a homing-vector instinct, a built-in policy of running directly toward a recently visited shelter. This system would produce the same result (homing vectors in conditions 2 and 3), and it better corresponds to known navigation strategies in rodent escape behavior.[20–22] Our implementation of the dual system switches to the rapid learner when the model-free learner fails to produce a valuable action. Previous work on dual-system arbitration has generated more sophisticated hypotheses, such as selecting the system with less uncertain action values[32] or with a history of reliably lower prediction errors.[45] Reliability-based arbitration may require an implausibly high number of practice runs. However, uncertainty-based arbitration should work with our results: assuming that the rewarded shelter state starts with very low prior uncertainty, this uncertainty should take longer to propagate back to the threat zone in conditions 2 and 3 than in condition 1.

The third condition modeled was the laser trip wire. With unlimited uniform exploration, the RL models found valid but convoluted escape routes around the trip wire. However, with a limited exploration period punctuated with practice edge-vector sequences, the on-policy SR agent learned escape routes in condition 1, but not in condition 3. Through the logic of the dual-system agent described above, this agent therefore invokes the backup homing-vector policy here, mirroring mouse behavior. This supports the notion that the mice's non-uniform exploratory paths—with runs to the shelter and obstacle edges being more rapid and direct than paths in the center and perimeter—is a crucial factor in modeling their spatial learning capabilities.[46]

One key difference from actual biological learning is the number of runs needed for learning: mice required 1–2 runs to learn the edge-vector route, while Q-learning and SR agents took tens of

practice runs. One possibility is that mice construct a value function or successor representation through a more data-efficient, model-based learning algorithm than the purely model-free updating mechanisms we used here.[29,47] Another possibility is that mice simply imbue certain actions (e.g. running toward salient objects) with a very high learning rate.[48] A final, compatible option is that they use a high-level representation of states and actions (e.g., "go from shelter area to obstacle edge" instead of "go north 10 cm") to speed up learning dramatically.[35] Indeed, agents that break down the arena into high-level regions and actions (e.g., a "threat-area-to-obstacle-edge" action) not only learned on a rapid timescale but also matched mice's capacity for spatial generalization in subgoal behavior. Unlike "flat" agents, operating at the level of individual grid-world states, this agent could execute edge-vector escapes after practicing nearby but non-identical routes. Hierarchical representations are known to allow for orders-of-magnitude increases in time and memory efficiency for planning, at the expense of overlooking routes that do not map directly onto the agent's high-level representation of the environment.[49] This state-action space also provides a straightforward explanation for our finding that subgoal escapes were selected based on spatial rules: the initiation set could correspond to a spatial region from which mice learned a valuable "go to obstacle edge" action. How animals might cluster states within their environments into these regions remains an interesting, open question.[49–51] This spatially sophisticated representation within model-free learning illustrates a disconnect between "map-based" and "model-based" methods. While we are invoking a spatial map to define states and actions, we do not need to invoke a model-based search through that map to uncover routes. Caching state-action values or a successor representation within a hierarchical spatial map is perfectly compatible with mouse escape trajectories.

A key remaining question is to define the scope of action-driven subgoal mapping. First, is the persistent subgoal strategy specific to escape behavior? Reactions to imminent threats tend to be less deliberate and flexible than less urgent behaviors such as reward seeking;[52] this raises the possibility that the persistent usage of memorized subgoals could be specific to escape. However, previous studies have also shown that rats[53] and mice[22] tend to prefer familiar routes over new shortcut routes even during reward seeking. This suggests that subgoal memorization is a general learning strategy across task modalities. Second, does action-driven mapping extend across species to human behavior? Clearly, an adult human in a small, well-lit room would not need to run to an obstacle edge in order to learn its location. However, humans may use analogous strategies in other scenarios. For example, De Cothi et al.[28] showed that in a virtual environment with changing obstacles and a limited visual field, humans tend to update their spatial behavior gradually based on the paths they take rather than immediately upon observing an obstacle. In addition, as in subgoal behavior, humans naturally break down multi-step tasks into high-level state and action representations.[50,54] For example, previous work has shown that human participants prefer paths that include sub-paths experienced during training, even if a shorter route was available.[49,55] Overall, it is highly plausible that action-driven mapping forms a part of the human cognitive repertoire. Future work across different species and behaviors will be needed to build a broader picture of the role of action-driven mapping in mammalian cognition at large.

STAR★METHODS

Detailed methods are provided in the online version of this paper and include the following:

- KEY RESOURCES TABLE
- RESOURCE AVAILABILITY
  - Lead contact
  - Materials availability
  - Data and code availability
- EXPERIMENTAL MODEL AND SUBJECT DETAILS
  - Animals
- METHOD DETAILS
  - Viral injection and fiber-optic cannula implantation
  - Behavioral apparatus
  - Closed-loop optogenetic stimulation
  - Exploration and escape behavior
  - Place preference assay
- QUANTIFICATION AND STATISTICAL ANALYSIS
  - Video tracking
  - Calculating position, speed and heading direction
  - Analysis of escape trajectories
  - Extraction of spontaneous homing runs and edge-vector runs
  - Initiation set analysis: Logistic regression
  - Initiation set analysis: Normalizing a metric
  - Initiation set analysis: Correlation analysis
  - Initiation set analysis: Testing for bias
  - Statistics
  - Reinforcement learning simulations

SUPPLEMENTAL INFORMATION

Supplemental information can be found online at https://doi.org/10.1016/j.neuron.2023.03.034.

ACKNOWLEDGMENTS

This work was funded by a Wellcome Senior Research Fellowship (214352/Z/18/Z), the Sainsbury Wellcome Centre Core Grant from the Gatsby Charitable Foundation and Wellcome (090843/F/09/Z) (T.B.), the Sainsbury Wellcome Centre PhD Programme (P.S.), and a Sir Henry Dale Fellowship from the Wellcome Trust and Royal Society (216386/Z/19/Z) (A.M.S.). A.M.S. is a CIFAR Azrieli Global Scholar in the Learning in Machines & Brains program. S.L. is supported by an EPSRC DTP Studentship as well as by the Gatsby Charitable Foundation (GAT3755) (SL/AS). We thank members of the Branco lab for discussions; Jesse Geerts, Marcus Stephenson-Jones, Caswell Barry, Catarina Albergaria, and Ted Moscovitz for comments on the manuscript; Panagiota Iordanidou for experimental support; and the Sainsbury Wellcome Centre Neurobiological Research Facility and FabLabs for technical support.

AUTHOR CONTRIBUTIONS

P.S. performed all experiments and data analysis. P.S. and S.L. performed theoretical modeling with supervision from A.M.S. P.S. and T.B. conceived the project, designed experiments, and co-wrote the manuscript.

CellPress
OPEN ACCESS

DECLARATION OF INTERESTS

The authors declare no competing interests.

Received: December 16, 2021
Revised: October 12, 2022
Accepted: March 27, 2023
Published: April 27, 2023

REFERENCES

1. Hull, C.L. (1934). The concept of the Habit-Family hierarchy, and maze learning. Part I. Psychol. Rev. *41*, 33–54.
2. Restle, F. (1957). Discrimination of cues in mazes: A resolution of the place-vs.-response question. Psychol. Rev. *64*, 217–228.
3. Tolman, E.C. (1948). Cognitive maps in rats and men. Psychol. Rev. *55*, 189–208.
4. O'Keefe, J., and Nadel, L. (1978). The Hippocampus as a Cognitive Map (Clarendon Press).
5. Doeller, C.F., King, J.A., and Burgess, N. (2008). Parallel striatal and hippocampal systems for landmarks and boundaries in spatial memory. Proc. Natl. Acad. Sci. USA *105*, 5915–5920.
6. Packard, M.G., Hirsh, R., and White, N.M. (1989). Differential effects of fornix and caudate nucleus lesions on two radial maze tasks: Evidence for multiple memory systems. J. Neurosci. *9*, 1465–1472.
7. Burgess, N., Recce, M., and O'Keefe, J. (1994). A model of hippocampal function. Neural Netw. *7*, 1065–1081.
8. Dordek, Y., Soudry, D., Meir, R., and Derdikman, D. (2016). Extracting grid cell characteristics from place cell inputs using non-negative principal component analysis. Elife *5*, e10094.
9. Foster, D.J., Morris, R.G., and Dayan, P. (2000). A model of hippocampally dependent navigation, using the temporal difference learning rule. Hippocampus *10*, 1–16.
10. McNamee, D.C., Stachenfeld, K.L., Botvinick, M.M., and Gershman, S.J. (2021). Flexible modulation of sequence generation in the entorhinal-hippocampal system. Nat. Neurosci. *24*, 851–862.
11. Cheng, K., Huttenlocher, J., and Newcombe, N.S. (2013). 25 years of research on the use of geometry in spatial reorientation: A current theoretical perspective. Psychon. Bull. Rev. *20*, 1033–1054.
12. Morris, R.G. (1981). Spatial localization does not require the presence of local cues. Learn. Motiv. *12*, 239–260.
13. Tolman, E.C., and Honzik, C.H. (1930). Introduction and removal of reward, and maze performance in rats. Publ. Psychol. *4*, 257–275.
14. Crowcroft, P. (1966). Mice All Over (G.T. Foulis & Co., Ltd.).
15. Alyan, S.H. (2004). Movement is necessary for landmark-based navigation. Belg. J. Zool. *134*, 61–63.
16. Ballard, D.H., Hayhoe, M.M., Pook, P.K., and Rao, R.P. (1997). Deictic codes for the embodiment of cognition. Behav. Brain Sci. *20*, 723–742.
17. Clark, A. (1999). An embodied cognitive science? Trends Cogn. Sci. *3*, 345–351.
18. Mataric, M.J. (1992). Integration of representation into goal-driven behavior-based robots. IEEE Trans. Rob. Autom. *8*, 304–312.
19. Evans, D.A., Stempel, A.V., Vale, R., and Branco, T. (2019). Cognitive control of escape behaviour. Trends Cogn. Sci. *23*, 334–348.
20. Vale, R., Evans, D.A., and Branco, T. (2017). Rapid spatial learning controls instinctive defensive behavior in mice. Curr. Biol. *27*, 1342–1349.
21. Maaswinkel, H., and Whishaw, I.Q. (1999). Homing with locale, taxon, and dead reckoning strategies by foraging rats: Sensory hierarchy in spatial navigation. Behav. Brain Res. *99*, 143–152.
22. Shamash, P., Olesen, S.F., Iordanidou, P., Campagner, D., Banerjee, N., and Branco, T. (2021). Mice learn multi-step routes by memorizing subgoal locations. Nat. Neurosci. *24*, 1270–1279.
23. Yilmaz, M., and Meister, M. (2013). Rapid innate defensive responses of mice to looming visual stimuli. Curr. Biol. *23*, 2011–2015.
24. Gradinaru, V., Thompson, K.R., Zhang, F., Mogri, M., Kay, K., Schneider, M.B., and Deisseroth, K. (2007). Targeting and readout strategies for fast optical neural control in vitro and in vivo. J. Neurosci. *27*, 14231–14238.
25. Magno, L.A.V., Tenza-Ferrer, H., Collodetti, M., Aguiar, M.F.G., Rodrigues, A.P.C., da Silva, R.S., Silva, J.d.P., Nicolau, N.F., Rosa, D.V., Birbrair, A., et al. (2019). Optogenetic stimulation of the M2 cortex reverts motor dysfunction in a mouse model of parkinson's disease. J. Neurosci. *39*, 3234–3248.
26. McFadden, D. (1977). Quantitative methods for analyzing travel behavior of individuals: Some recent developments. Cowles Foundation Discussion Papers707.
27. Burnham, K.P., and Anderson, D.R. (2004). Multimodel inference: Understanding AIC and BIC in model selection. Sociol. Methods Res. *33*, 261–304.
28. De Cothi, W., Nyberg, N., Griesbauer, E.-M., Ghanamé, C., Zisch, F., Lefort, J.M., Fletcher, L., Newton, C., Renaudineau, S., Bendor, D., et al. (2022). Predictive maps in rats and humans for spatial navigation. Curr. Biol. *32*, 3676–3689.e5.
29. Russek, E.M., Momennejad, I., Botvinick, M.M., Gershman, S.J., and Daw, N.D. (2017). Predictive representations can link model-based reinforcement learning to model-free mechanisms. PLoS Comput. Biol. *13*, e1005768.
30. Dayan, P. (1993). Improving generalization for temporal difference learning: The successor representation. Neural Comput. *5*, 613–624.
31. Geerts, J.P., Chersi, F., Stachenfeld, K.L., and Burgess, N. (2020). A general model of hippocampal and dorsal striatal learning and decision making. Proc. Natl. Acad. Sci. USA *117*, 31427–31437.
32. Daw, N.D., Niv, Y., and Dayan, P. (2005). Uncertainty-based competition between prefrontal and dorsolateral striatal systems for behavioral control. Nat. Neurosci. *8*, 1704–1711.
33. Sutton, R.S., and Barto, A.G. (2018). Reinforcement Learning: An Introduction (MIT Press).
34. Sutton, R.S. (1995). Generalization in reinforcement learning: Successful examples using sparse coarse coding. In Advances in Neural Information Processing Systems, *8*, D. Touretzky, M.C. Mozer, and M. Hasselmo, eds..
35. Sutton, R.S., Precup, D., and Singh, S. (1999). Between MDPs and semi-MDPs: A framework for temporal abstraction in reinforcement learning. Artif. Intell. *112*, 181–211.
36. Edvardsen, V., Bicanski, A., and Burgess, N. (2020). Navigating with grid and place cells in cluttered environments. Hippocampus *30*, 220–232.
37. Spiers, H.J., and Gilbert, S.J. (2015). Solving the detour problem in navigation: A model of prefrontal and hippocampal interactions. Front. Hum. Neurosci. *9*, 125.
38. Schulz, E., and Gershman, S.J. (2019). The algorithmic architecture of exploration in the human brain. Curr. Opin. Neurobiol. *55*, 7–14.
39. Schulz E., Klenske E.D., Bramley N.R., Speekenbrink M. Strategic exploration in human adaptive control. 2017. Preprint at bioRxiv. 10.1101/110486.
40. Ward, D., Silverman, D., and Villalobos, M. (2017). Introduction: The varieties of enactivism. Topoi *36*, 365–375.
41. Chase, W.G., and Simon, H.A. (1973). Perception in chess. Cogn. Psychol. *4*, 55–81.
42. Petitto, L.A., and Marentette, P.F. (1991). Babbling in the manual mode: Evidence for the ontogeny of language. Science *251*, 1493–1496.
43. Gollub, L. (1977). Conditioned reinforcement: Schedule effects. In Handbook of Operant Behavior, W.K. Honig and J.E.R. Staddon, eds. (Routledge), pp. 288–312.

44. Baron, S.P., and Meltzer, L.T. (2001). Mouse strains differ under a simple schedule of operant learning. Behav. Brain Res. *118*, 143–152.

45. Lee, S.W., Shimojo, S., and O'Doherty, J.P. (2014). Neural computations underlying arbitration between model-based and model-free learning. Neuron *81*, 687–699.

46. McNamee, D.C., Stachenfeld, K.L., Botvinick, M.M., and Gershman, S.J. (2021). Flexible modulation of sequence generation in the entorhinal–hippocampal system. Nat. Neurosci. *24*, 851–862.

47. Sutton, R.S. (1991). Dyna, an integrated architecture for learning, planning, and reacting. SIGART Bull. *2*, 160–163.

48. Barto, A.G., Singh, S., and Chentanez, N. (2004). Intrinsically motivated learning of hierarchical collections of skills. In Proceedings of the 3rd International Conference on Development and Learning, J. Triesch and T. Jebara, eds. (UCSD Institute for Neural Computation), pp. 112–119.

49. Tomov, M.S., Yagati, S., Kumar, A., Yang, W., and Gershman, S.J. (2020). Discovery of hierarchical representations for efficient planning. PLoS Comput. Biol. *16*, e1007594.

50. Solway, A., Diuk, C., Córdova, N., Yee, D., Barto, A.G., Niv, Y., and Botvinick, M.M. (2014). Optimal behavioral hierarchy. PLoS Comput. Biol. *10*, e1003779.

51. Stachenfeld, K.L., Botvinick, M.M., and Gershman, S.J. (2017). The hippocampus as a predictive map. Nat. Neurosci. *20*, 1643–1653.

52. Mobbs, D., Headley, D.B., Ding, W., and Dayan, P. (2020). Space, time, and fear: Survival computations along defensive circuits. Trends Cogn. Sci. *24*, 228–241.

53. Grieves, R.M., and Dudchenko, P.A. (2013). Cognitive maps and spatial inference in animals: Rats fail to take a novel shortcut, but can take a previously experienced one. Learn. Motiv. *44*, 81–92.

54. Ribas-Fernandes, J.J.F., Solway, A., Diuk, C., McGuire, J.T., Barto, A.G., Niv, Y., and Botvinick, M.M. (2011). A neural signature of hierarchical reinforcement learning. Neuron *71*, 370–379.

55. Huys, Q.J., Lally, N., Faulkner, P., Eshel, N., Seifritz, E., Gershman, S.J., Dayan, P., and Roiser, J.P. (2015). Interplay of approximate planning strategies. Proc. Natl. Acad. Sci. USA *112*, 3098–3103.

56. Allen Institute for Brain Science (2015). Allen Mouse Brain Atlas. https://mouse.brain-map.org/static/atlas.

57. Shamash, P., Carandini, M., Harris, K., and Steinmetz, N. (2018). A tool for analyzing electrode tracks from slice histology. Preprint at bioRxiv. https://doi.org/10.1101/447995.

58. Lopes, G., Bonacchi, N., Frazão, J., Neto, J.P., Atallah, B.V., Soares, S., Moreira, L., Matias, S., Itskov, P.M., Correia, P.A., et al. (2015). Bonsai: An event-based framework for processing and controlling data streams. Front. Neuroinform. *9*, 7.

59. Shamash, P., and Branco, T. (2022). Protocol to study spatial subgoal learning using escape behavior in mice. Bio. Protoc. *12*, e4443.

60. Stamatakis, A.M., and Stuber, G.D. (2012). Activation of lateral habenula inputs to the ventral midbrain promotes behavioral avoidance. Nat. Neurosci. *15*, 1105–1107.

61. Schlesinger, K., Lipsitz, D.U., Peck, P.L., Pelleymounter, M.A., Stewart, J.M., and Chase, T.N. (1983). Substance P enhancement of passive and active avoidance conditioning in mice. Pharmacol. Biochem. Behav. *19*, 655–661.

62. Mathis, A., Mamidanna, P., Cury, K.M., Abe, T., Murthy, V.N., Mathis, M.W., and Bethge, M. (2018). Deeplabcut: Markerless pose estimation of user-defined body parts with deep learning. Nat. Neurosci. *21*, 1281–1289.

63. Watkins, C.J., and Dayan, P. (1992). Technical note: Q-learning. Mach. Learn. *8*, 279–292.

64. Gershman, S.J., Moore, C.D., Todd, M.T., Norman, K.A., and Sederberg, P.B. (2012). The successor representation and temporal context. Neural Comput. *24*, 1553–1568.

65. Hart, P., Nilsson, N., and Raphael, B. (1968). A formal basis for the heuristic determination of minimum cost paths. IEEE Trans. Syst. Sci. Cyber. *4*, 100–107.

STAR★METHODS

KEY RESOURCES TABLE

REAGENT or RESOURCE	SOURCE	IDENTIFIER
Experimental models: Organisms/strains		
Mouse: c57bl6	Charles Rivers	N/A
Software and algorithms		
Software to run escape and laser trip wire experiments in bonsai	Zenodo repository	https://doi.org/10.5281/zenodo.7677414
Python package for analyzing free-moving behavioral data during optogenetics experiments	Zenodo repository	https://doi.org/10.5281/zenodo.7677429
Reinforcement learning agents escaping to shelter with a barrier	Zenodo repository	https://doi.org/10.5281/zenodo.7677456

RESOURCE AVAILABILITY

Lead contact

Further information and requests for resources and reagents should be directed to and will be fulfilled by the lead contact, Tiago Branco (t.branco@ucl.ac.uk).

Materials availability

This study did not generate new unique reagents.

Data and code availability

The data reported in this paper will be shared by the lead contact upon request. All original code has been deposited at Zenodo and is publicly available as of the date of publication. DOIs are listed in the key resources table. Any additional information required to re-analyze the data reported in this paper is available from the lead contact upon request.

The data-acquisition software is available from Github: https://github.com/philshams/bonsai-behavior, the data-analysis software is available from Github: https://github.com/philshams/behavior-opto-analysis, and the RL simulation software is available from Github: https://github.com/philshams/Euclidean_Gridworld_RL. The data from this study will be made available upon publication.

EXPERIMENTAL MODEL AND SUBJECT DETAILS

Animals

All experiments were performed under the UK Animals (Scientific Procedures) Act of 1986 (PPL70/7652) after local ethical approval by the Sainsbury Wellcome Center Animal Welfare Ethical Review Body. We used 36 singly housed (starting from 8 weeks old), male, 8–12-week-old C57BL/6J mice (Charles River Laboratories) during the light phase of the 12-h light/dark cycle. Mice were housed at 22°C and in 55% relative humidity with *ad libitum* access to food and water.

Re-use over multiple sessions

For the exploration and escape experiments in implanted mice (experiments 1–6): four of the eight mice were naive, and this was their first behavioral session. The remaining four mice had experienced a previous session 5–7 days prior. Their previous session was not allowed to be the same exact experiment as the second session but was otherwise selected randomly. The effects of having a previous session on escape behavior were modest (Figure S2E-F), and do not impact the interpretation of our results. For the place-preference experiment and laser-power test, mice were randomly selected from those that had already experienced their behavioral sessions in experiments 1–6. For the experiments in unimplanted mice, experiment #7 was performed in naive mice, and experiment #8 was performed 5–7 days later, with the same set of mice.

Exclusion criteria

Data from mice with zero escapes in the session (three mice: due to staying in the shelter; two mice: due to not responding to the threat stimulus; one mouse: due to climbing down from the platform; all mice had a previous session) were excluded, and a replacement session was performed 5–7 days later in a randomly selected mouse.

METHOD DETAILS

Viral injection and fiber-optic cannula implantation

Surgical procedure

Mice were anesthetized with isoflurane (5%) and secured on a stereotaxic frame (Kopf Instruments). Meloxicam was administered subcutaneously for analgesia. Isoflurane (1.5–2.5% in oxygen, 1 L min−1) was used to maintain anesthesia. Craniotomies were made using a 0.7 mm burr (Meisinger) on a micromotor drill (L12M, Osada), and coordinates were measured from bregma. Viral vectors were delivered using pulled glass pipettes (10 μL Wiretrol II pulled with a Sutter-97) and an injection system coupled to a hydraulic micromanipulator (Narishige), at approximately 100 nL min−1. Implants were affixed using light-cured dental cement (3M) and the surgical wound was closed using surgical glue (Vetbond).

Injection and implantation

Mice were injected with 120 nL of AAV9/CamKIIa-ChR2-EGFP in the right, anterior premotor cortex (AP: 2.4 mm, ML: 1.0 mm, DV: −0.75 mm relative to brain surface) and implanted with a magnetic fiber-optic cannula directly above the viral injection (DV: −0.5 mm) (MFC_200/245–0.37_1.5mm_SMR_FLT, Doric). All behavioral sessions took place 2–4 weeks after the injection/implantation.

Histology

To confirm injection and implantation sites, mice were terminally anesthetized by pentobarbital injection and decapitated for brain extraction. The brains were left in 4% PFA overnight at 4°C. 100um-thick coronal slices were acquired using a standard vibratome (Leica). The sections were then counter-stained with 4′,6-diamidino-2-phenylindole (DAPI; 3 μM in PBS), and mounted on slides in SlowFade Gold antifade mountant (Thermo Fisher, S36936) before imaging (Zeiss Axio Imager 2). Histological slice images were registered to the Allen Mouse Brain Atlas[56] using SHARP-Track,[57] to find the fiber tip coordinates.

Behavioral apparatus

Platform and shelter

Experiments took place on an elevated white 5-mm-thick acrylic circular platform 92 cm in diameter. The platform had a 50 × 10 cm rectangular gap in its center. For conditions with no obstacle (all post-exploration escapes and the entirety of experiments 3 and 10), this was filled with a 50 × 10 cm white 5-mm-thick acrylic rectangular panel (Figure S2B). For conditions with the obstacle present (the exploration period in experiments 1–2 and 4–8), this was filled with an identical panel that, attached to an obstacle: a 50 cm long x 12.5 cm tall x 5 mm thick white acrylic panel (Figure S2A). The shelter was 20 cm wide x 10 cm deep x 15 cm tall and made of 5-mm-thick transparent red acrylic, which is opaque to the mouse but transparent to an infrared-detecting camera. The shelter had a 9cm-wide entrance at the front, which extended up to the top of the shelter and then 5 cm along its ceiling; this extension of the opening allowed the optic fiber, which was plugged into the mouse's head, to enter the shelter without twisting or giving resistive force.

Additional hardware

The elevated platform was in a 160 cm wide x 190 cm tall x 165 cm deep sound-proof box. A square-shaped projector screen (Xerox) was located above the platform. This screen was illuminated in uniform, gray light at 5.2 cd m^{-2} using a projector (BenQ). Behavioral sessions were recorded with an overhead GigE camera (Basler) with a near-infrared selective filter, at 40 frames per second. Six infrared LED illuminators (TV6700, Abus) distributed above the platform illuminated it for infrared Video recording. All signals and stimuli, including each camera frame, were triggered and synchronized using hardware-time signals controlled with a PCIe-6351 and USB-6343 input/output board (National Instruments), operating at 10 kHz. The platform and shelter were cleaned with 70% ethanol after each session.

Data acquisition software and online video tracking

Data acquisition was performed using custom software in the visual reactive programming language Bonsai.[58] In order to automatically deliver laser and auditory stimuli (see below), mice were tracked online during each behavioral session. Online tracking was based on the mouse being darker than the white acrylic platform; we used the following Bonsai functions, in this order: BackgroundSubtraction, FindContours, BinaryRegionAnalysis, and LargestBinaryRegion.

Closed-loop optogenetic stimulation

Laser stimuli consisted of 2-s, 20-HZ square-wave pulses at 30 mW (duty cycle 50%, so 15 mW average power over the 2 s) supplied by a 473-nm laser (Stradus 472, Vortran). For experiment #5, we instead used 5-s pulses. The laser was controlled by an analog signal from our input/output board into the laser control box. At the beginning of each session, the mouse was placed in an open 10 × 10 cm box and the magnetic fiber-optic cannula was manually attached to a fiber-optic cable (MFP_200/230/900_0.37_1.3m_FC-SMC, Doric). A rotary joint (Doric) was connected to the laser via a 200-μm core patch cable (ThorLabs) and used to prevent the cable from twisting. At the beginning of each mouse's first session, the mouse was placed in a 10 × 10 cm box, and two 2-s stimuli were applied. If these did not evoke stopping and leftward turning (2/24 mice), then the mouse was assigned to one of the laser-off conditions (experiment 1 or 3). During laser-on sessions, the criteria for triggering laser stimuli were: 1) the mouse crosses the 'trip wire' (illustrated in Figures 1, 3, 4); and 2) the mouse is moving in the 'correct' direction. For blocking edge-vector and edge-to-shelter runs, the direction was determined by a directional speed threshold: moving toward the shelter area (i.e., south) at > 5 cm s^{-1}. For blocking threat-zone-to-left-side runs, mice had to be moving toward the left side (i.e., west) at > 5 cm s^{-1}. These

speed thresholds are low enough to be effective at catching all cases in which the mouse crosses the trip wire in a particular direction. These criteria were computed online using the Bonsai software described in the previous section. The laser pulses were emitted with a delay of 300–400 ms after being triggered. Up to three subsequent 2-s pulses (or one 5-s pulse in experiment #5) were triggered manually if the mouse continued moving forward. Mice usually took 1-3 min to enter the shelter for the first time, and these first minute(s) of exploration typically contains relatively vigorous running. Since subgoal learning does not occur in this setting without a shelter in the environment,[22] the laser-on condition was initiated only after the mouse entered the shelter for the first time.

Exploration and escape behavior

A list of the different experimental configurations is given in the below table.

List of all experiments

ID	Experimental setup	M2 stimulation	Mice	Figures
1	obstacle removal	injection/implantation, no stim	8	Figures 1, 2, S3, S4, and S7
2	obstacle removal	stop edge-vector runs	8	Figures 1, 2, S1, S3, and S4
3	open field—no obstacle	injection/implantation, no stim	8	Figures 2 and S4
4	obstacle removal	stop edge-vector runs after two	8	Figures S5–S7
5	obstacle removal	stop edge-to-shelter runs	8	Figures 3, S6, and S7
6	obstacle removal	stop threat-area-to-left-side runs	8	Figures 4, S6, and S7
7	obstacle removal—threat zone II	none	8	Figure S7
8	obstacle removal—threat zone III	none	8	Figures 4 and S7
9	two-chamber place preference	paired with one chamber	8	Figure S1
10	open field—no obstacle or shelter	test effects of three laser powers	4	Figure S1

Auditory threat stimuli

Threat stimuli were loud (84 dB), unexpected crashing sounds played from a speaker located 1 m above the center of the platform (Data S1). Sounds ('smashing' and 'crackling fireplace') were downloaded from soundbible.com. They were then edited using Audacity 2.3.0, such that they were 1.5 s long and continuously loud. Stimuli alternated between the 'smashing' sound and the 'crackling' sound each trial, to prevent stimulus habituation. The volume was increased by 2 dB after time a stimulus failed to elicit an escape, up to a maximum of 88 dB. When a threat trial began, the stimuli repeated until the mouse reached the shelter or for a maximum of 9 s.

Triggering escapes

The criteria for activating a threat stimulus were 1) the mouse is currently in the threat zone (illustrated in Figure 2); 2) the mouse was in the threat zone 1.5 s ago; 3) the mouse is moving away from the shelter at >5 cm s^{-1} (this ensures that escape runs are always initiated after the stimulus onset); 4) the most recent threat stimulus occurred >45 s ago. These criteria were computed online using the Bonsai software described above, and auditory threat stimuli played automatically when all four criteria were met. Experiments were terminated after six successful escapes or 1 h. In experiments 7–9, criterion #2 was not applied. For experiment #8, experiments were terminated after ten escapes rather than six, as this threat zone allowed for more trials. Reaching the shelter was defined as reaching any point within 10 cm of the shelter entrance, and escapes were considered successful if they reached the shelter within the 9-s stimulus period.

Obstacle removal

After 20 min of exploration were complete, as soon as the mouse entered the shelter, the experimenter quickly and quietly removed the central panel containing the obstacle and replaced it with the flat 50 × 10 cm panel. Mice were then allowed to freely explore and (and trigger escapes) in this open-field platform.

Exploration time

Mice were given 10 min of exploration in the open arena before the threat zone became active. This provides enough time for the mice to locate the shelter and adopt the shelter as their home base. In the arena with the obstacle, mice had 20 min of exploration, allowing enough time to additionally perform edge-vector runs and learn subgoals. The threat zone then became active immediately after the obstacle was removed. Since this condition does not allow for much time to explore the obstacle-free environment before facing threat stimuli, we found that the shorter exploration time (10 min) in the open arena provides a fairer comparison.

Adding bedding to the platform

Bedding from the mouse's home cage was added to the platform in order to encourage exploration, rather than staying in the shelter throughout the experiment. One pinch (~1g) of bedding was added to the center of the threat zone in all experiments when either of the following two criteria was met: 1) The mouse did not leave the shelter for 5 min; or 2) The mouse did not enter the threat zone for 10 min. In order to encourage occupancy of the areas from which edge-vector runs initiate, a pinch of bedding was placed on the left

side of the threat zone in experiments #4 and 6, and the left and right sides in experiments 7–8. In order to maintain comparability across conditions, a pinch of bedding was also placed in the same location for the mice with a previous session in experiment #2. See Shamash and Branco[59] for a step-by-step behavioral protocol.

Place preference assay

Mice were hooked up to the optic fiber as described above and placed into a two-chamber place preference arena. The arena was made of 5-mm-thick transparent red acrylic (opaque to the mouse) and consisted of two 18 cm long x 18 cm wide x 18 cm tall chambers connected by a 8cm-long opening. To make the chambers visually distinguishable, one chamber had a 10 × 10 cm x-shaped white acrylic piece affixed to its back wall and the other had a filled-in, 10cm-diameter circular white acrylic piece affixed to its back wall. The stimulation chamber (left or right) was pseudoramdomly determined before each session, such that both sides ended up with four mice. After a 1-min habituation period, a series of four 2-s laser stimuli were manually triggered whenever the mouse fully entered the stimulation chamber. A minimum of 1 min was given in between each trial, and a total of six stimulation series were delivered. After the last stimulation, 1 min was given so that the occupancy data would not be biased by always starting in the stimulation chamber. Then, the next 20 min were examined to test for place aversion in the stimulation chamber. This assay is adapted from the conditioned place preference assay[60] and the passive place avoidance assay,[61] such that it matches the conditions of our exploration/escape assay (i.e., to be relevant, place aversion must be elicited during the same session as the laser stimulation, and it must be expressed through biases in occupancy patterns).

QUANTIFICATION AND STATISTICAL ANALYSIS

All analysis was done using custom software written in Python 3.8 as well as open-source libraries, notably NumPy, OpenCV, Matplotlib and DeepLabCut. See Shamash and Branco[59] for additional details on quantification of escape trajectories.

Video tracking

Video recording was performed with custom software in Bonsai. We used DeepLabCut[62] to track the mouse from the Video, after labeling 412 frames with 13 body parts: snout, left eye, right eye, left ear, neck, right ear, left upper limb, upper back, right upper limb, left hindlimb, lower back, right hindlimb and tail base (Video 5). Post-processing includes removing low-confidence tracking, using a median filter with a width of 7 frames and applying a linear transformation to the tracked coordinates to match all Videos to the same coordinate reference frame. Videos were generated using custom Python code, the OpenCV library and Adobe AfterEffects.

Calculating position, speed and heading direction

For analysis of escape trajectories and exploration, we used the average of all 13 tracked points, which we found to be more stable and consistent than any individual point. To calculate speed, we smoothed the raw frame-by-frame speed with a Gaussian filter ($\sigma = 4$ frames = 100 ms). To calculate the mouse's body direction, we computed the vector between the lower body (averaging the lower left limb, lower right limb, lower back, and tail base) and the front of the body (averaging the upper left limb, upper right limb, and upper back). See Video 5 for a visualization of the tracking and of these calculations.

Analysis of escape trajectories

The escape target score was computed by taking the vector from the mouse's position at escape initiation to its position when it was 10 cm in front of the obstacle. Vectors aimed directly at the shelter received a value of 0; those aimed at the obstacle edge received a value of 1.0; a vector halfway between these would score 0.5; and a vector that points beyond the edge would receive a value greater than 1.0. The formula is:

$$score = \frac{|offset_{HV} - offset_{EV} + offset_{HV-EV}|}{2 * offset_{HV-EV}}$$

OffsetHV is the distance from the mouse to where the mouse would be if it took the homing vector; offsetEV is the distance from the mouse to where the mouse would be if it took the obstacle edge vector; and offsetHV −EV is the distance from the homing vector path to the obstacle edge vector path. The threshold for classifying a trajectory as an edge vector (scores above 0.65) was taken from Shamash et al.,[22] where it represented the 95th percentile of escapes in the open-field condition. Escapes with scores under 0.65 were designated as homing vectors. When escape trajectories are limited to escapes on the left side, this refers to escapes that are on the left half of the arena when they cross the center of the platform along the vertical (threat-shelter) axis.

The escape initiation point occurs when mice surpass a speed of 20 cm s^{-1}, relative to (i.e., getting closer to) the shelter location. This threshold is high enough to correctly reject non-escape locomotion bouts along the perimeter of the platform but also low enough to identify the beginning of the escape trajectory.

Extraction of spontaneous homing runs and edge-vector runs

Homing runs are continuous turn-and-run movements from the threat area toward the shelter and/or obstacle edges. As in Shamash et al.,[22] they are extracted by (1) computing the mouse's 'homing speed' (that is, speed with respect to the shelter or obstacle edges with Gaussian smoothing (/sigma = 0.5 s)) and the mouse's 'angular homing speed' (the rate of change of heading direction with respect to the shelter or obstacle edges); (2) identifying all frames in which the mouse has a homing speed of >15 cm s^{-1} or is turning toward the shelter at an angular speed of >90° per s; (3) selecting all frames within 1 s of these frames, to include individual frames that might be part of the same homing movement but do not meet the speed criteria; (4) rejecting all frames in which the mouse is not approaching or turning toward an edge or the shelter; and (5) rejecting sequences that take less than 1 s or do not decrease the distance to the shelter by at least 20%. Each series of frames that meet these criteria represents one homing run. We limited analysis to the homing runs that started within the threat area (Figure 1A). Edge-vector runs are homing runs that enter anywhere within the 10-cm-long (along the axis parallel to the obstacle) × 5-cm-wide (along the axis perpendicular to the obstacle) rectangle centered 2.5 cm to the left of the obstacle edge.

Initiation set analysis: Logistic regression

Our logistic regression analysis tests the strength of the linear relationship between each spatial metric and the log odds of performing an edge-vector escape. No regularization penalty was used. The strength of the fit was measured using McFadden's pseudo-R^2 $R^2 = 1 - \frac{LL_{full}}{LL_{null}}$, where LLfull is the log likelihood of the logistic regression model fitted with the predictor data and LLnull is the log likelihood of the logistic regression fitted with only an intercept and no predictor data. Pseudo-R^2 values of 0.2–0.4 represent "excellent fit".[26] To test statistical significance of these values, we performed a permutation test, based on the distribution of pseudo-R^2 for the same predictor value, across 10,000 random shuffles of the escape responses (edge vector or homing vector).

Initiation set analysis: Normalizing a metric

To normalize a spatial metric (y, e.g. distance from the center of the arena along the left-right axis) by another metric (x, e.g. distance from the shelter), we computed a linear regression on these variables. We then took the residuals of this prediction (residual = y − y^, where y^ = slope × x + offset) and correlated them with proportion of edge vector escapes in each bin. This tells us whether, at a given distance from the shelter, there is still a correlation with distance from the center.

Initiation set analysis: Correlation analysis

To better visualize the relationship between the mouse's initial position and the likelihood of executing an edge-vector escape, we binned the spatial metric and computed the correlation to the proportion of edge-vectors in each bin. The widest possible range of values was selected, given the constraints that this range starts and ends on a multiple of 2.5 cm and that all bins contain at least six escapes. From this range, seven equal-sized bins were used. The correlation results were robust to the number of bins used.

Initiation set analysis: Testing for bias

To test whether correlations between edge-vector likelihood and spatial location could be the result of biases in the edge-vector classification computation, we performed a simulation analysis of escapes from throughout the threat zone, testing whether edge vector likelihood varied due to the escape's start point. The simulated escape routes followed a Von Mises distribution of vectors with a direction between the shelter and left obstacle edge. We used a distribution centered upon the direction 60% of the way from the homing-vector path to the edge vector path, corresponding to the mean target score of 0.6 in the obstacle removal experiments. The Von Mises distribution had a kappa value of 8.0, producing 50% edge vectors overall, corresponding to the proportion of edge vectors in the obstacle removal experiments. We simulated 100 escape trials starting from each square cm of threat zone (1652 total starting location). Thus, in each starting point, the simulated mice randomly selected from the average observed distribution of escape movements. We then examined whether there was any correlation between the average probability of an edge vector escape in each square-cm bin and the spatial location of the bin, similar to our analysis of the mouse escape data. In the mouse data, we observed that mice tended to execute more edge-vector escapes further from the central vertical axis and further from the obstacle. In the simulated data, there was a slight negative correlation between the distance from the central axis and the proportion of edge vectors (r = −0.16, p = 1 × 10−10, Pearson correlation). This is in the opposite direction of the observed trend in mice. In the other axis (distance from obstacle), there was no correlation between spatial location and edge-vector probability (r = 0.02, p = 0.4). We conclude that the spatial effects we saw were not due to bias in the metric.

Statistics

For comparisons between groups, we used a permutation test with the test statistic being the pooled group mean difference. The condition of each mouse (e.g., laser-on vs. laser-off) is randomly shuffled 10,000 times to generate a null distribution and a p value. We used this test because it combines two advantages: 1) Having the test statistic as the pooled group mean gives weight to each trial rather than collapsing each animal's data into its mean (as in the t-test or the Mann–Whitney test); 2) It is non-parametric and does not assume Gaussian noise (unlike the repeated-measures ANOVA), in line with much of our data. Tests for increases or decreases (e.g., whether exploration decreased due to laser stimulation) were one tailed. The Wilcoxon signed-rank test was used for the place-preference assay to test whether occupancy in the stimulation chamber was less than 50%. The sample size of our experiments (n = 8

mice) was selected based on a power analysis based on the data from Shamash et al. 2021 and a minimum power of 0.8. Ranges in boxplots are limited from the first quartile minus 1.5 x IQR to the third quartile plus 1.5 x IQR. Statistically significant results are indicated in the figures using the convention n.s.: $p > 0.05$, *: $p < 0.05$, **: $p < 0.01$ and ***: $p < 0.001$.

Reinforcement learning simulations

General reinforcement learning setup

Reinforcement learning simulations use the formalism of a Markov Decision Process (MDP).[33] An MDP consists of a tuple (S,A,T,R) where S is the set of states; A is the set of possible actions; $T: S \times A -> S'$ is the transition function defining what happens when an action α is taken in state s; $R: S \times A \times S' -> R$ is the reward function, which determines the scalar reward returned by the environment after a given state-action-next-state sequence. We construct our environment as a 13x13 gridworld. S consists of the set of accessible positions in this map, shown in white in the figures. A, unless stated otherwise, consists of 8 actions (north, northwest, west, southwest, south, southeast, east, northeast). T is a deterministic function that moves the agent one unit in the direction of the action taken. R is a deterministic function in which a reward of 100 is given for entry to the shelter state, and a negative reward of $d(s,s')$ is given for each transition. $d(s,s')$ is the distance between a pair of states s and s' - 1.0 for side-by-side states and $\sqrt{2}$ for diagonally separated states; using this negative reward is the mechanism by which the agents take sideways actions (north, west, etc.) to be shorter than diagonal actions (northwest, etc.). This negative reward was not present when the shelter was not in the environment, i.e. the training phase of condition 2, to avoid accumulating unmitigated negative value in each state-action pair.

In general, the reinforcement learning problem is to find a policy, π, which maps states to actions, such that the expected sum of discounted future rewards is maximized.[33]

$$E\left[\sum_{t=0}^{\infty} \gamma^t R_{a_t}(s_t, s_{t+1}) | S_0 = S\right]$$

where $a_t = \pi(s_t)$, i.e. actions given by the policy and γ is the temporal discount factor, a hyperparameter specifying how much long-term reward should be weighted against short-term reward. Each of the RL agents described below operates by searching for a policy that can optimize expected future reward. The algorithms have different limitations and compute their policies differently; thus, different algorithms often generate different policies. We compared the behavior of these various algorithms to mouse behavior, in order to end up with a concrete, computational description of mouse behavior.

Simulation details

Simulation experiments consisted of three phases: a training phase, a pre-test phase, and a test phase. Each algorithm was repeated 100 times with 100 different random seeds. Each agent started by being dropped in at a (uniform) random location in the arena. In the training phase, unless otherwise stated, the RL agent then moved around the environment with a random policy (probability of 1/8 for each action) and learned based on this experience. Moving into a barrier (black) resulted in the agent remaining in the same state from which it initiated an action in the previous timestep. Trip wires acted like barriers but only when the agent was attempting to pass the trip wire in the threat-area-to-obstacle-edge direction. Each algorithm received enough training steps that all 100 seeds was able to learn an escape to shelter in condition 1, after being dropped into the threat zone, rounded up to the nearest 500 steps (for models that took <10k steps) or 5k steps (for models that took >10k steps) (see below table). Thus, we are modeling only the mice that learn edge-vector escapes during the training phase. This number of training steps was used across all four conditions. In the pre-test phase, the agent started in the shelter and then moved randomly through the environment until reaching the threat zone square (learning was allowed to continue during this period). At this point, the test phase was initiated. The agent then stopped moving randomly and adopted its learned policy in order to navigate to the reward. After this a second and third trial (pre-test + test phase for each one) were performed. The test phase proceeded until the agent reached the shelter or for a maximum of 100 steps.

Hyperparameters

Algorithm	Hyperparameter	Value
Q-learning	temporal discount factor γ	0.9
Q-learning	TD(λ) decay factor γ	0.5
Q-learning	learning rate, α	0.1
Q-learning	neg. reward per step	0.01
SR	temporal discount factor γ	0.9
SR	TD(λ) decay factor γ	0.5
SR	learning rate, α	0.1
SARSA	temporal discount factor γ	0.99
SARSA	TD(λ) decay factor γ	0.5

(Continued on next page)

Continued

Algorithm	Hyperparameter	Value
SARSA	learning rate, α	0.1
SARSA	neg. reward per step	0.001
Tile coding	tile size	[2 × 2, 3 × 3]
MB-G	model buffer window, N	15

Hyper-parameters

In machine learning, and reinforcement learning in particular, models can be highly sensitive to hyper-parameters. Different hyper-parameter configurations can lead to different behavior even for the same algorithm. In the tabular setting, these sensitivities are well understood, but are nonetheless present (see ch. 2, ch.8 of Sutton and Barto[33]). While we did not conduct extensive comparison over hyper-parameters, we endeavored to use comparable settings across models and chose from typical ranges for grid-world environments in the RL literature (e.g. https://github.com/karpathy/reinforcejs). Some hyper-parameters, such as the initialization scheme in the value-based and successor-representation models, are particularly significant for learning speed, making it difficult to meaningfully calibrate learning speed across models. In general, due to the possibility of behavior changing across hyper-parameters, we are careful not to point to any one algorithm as ‘the match’ to mouse behavior; instead, we investigate the causes of behavior across a variety of algorithms in order to extract overarching computational principles.

Q-learning

At test time, the Q-learning agent generates a policy by selecting the action a in the current state s that has the maximum state-action value. State-action values are incrementally learned during the training and pre-test phases using the Q-learning algorithm[63] combined with an eligibility trace.[33] The eligibility trace is a decaying trace of recent state-action pairs. After taking action a_t in state s_t and moving to state s_{t+1}, the agent takes three steps to update its state-action values. First, it decays its eligibility trace e, by $e \leftarrow \lambda\gamma e$, where λ is the eligibility trace decay parameter and γ is the temporal discount factor introduced above. Second, it updates its eligibility trace to add the current state-action pair: $e(s_t,a_t) \leftarrow e(s_t,a_t) + 1$. Finally, it updates its state-action-value table:

$$Q(s_t,a_t) \leftarrow Q(s,a) + \alpha\left[r_t + \gamma \max_a Q(s_{t+1},a) - Q(s_t,a_t)\right]e$$

where r_t is the reward gained from this step, α is the learning rate and γ is the temporal discount factor. State-action values are initialized randomly with mean 0 and variance 0.1.

Tile coding

One limitation of tabular methods is that they are unable to generalize. Learning information (e.g. about value) in one state does not provide information about any other states. A common way to overcome this is to use function approximation to represent quantities rather than storing them explicitly in look-up tables. Among the simplest forms of function approximation is a linear map. For example, the approximate state-action value function can be defined as

$$\widehat{Q}(s,a,\mathbf{w}) \equiv \mathbf{w} \cdot \mathbf{x} = \sum_{i=1}^{d} w_i x_i(s,a)$$

where $\mathbf{x}$ is the featured state with dimension d, and $\mathbf{w}$ are learnable weights. The update rule for these weights under stochastic gradient descent is given by

$$\mathbf{w}_{t+1} = \mathbf{w}_t + \alpha\left[r_{t+1} + \gamma \max_{a'} Q(s',a') - Q(s,a)\right]Q(s,a)$$

where α is the learning rate and γ is the discount factor. One popular way to featurize a state space for linear methods is tile coding. The feature map consists of a set of overlapping receptive fields; for each field a state is said to be present—and given a feature value of 1—if it is within the receptive field, and absent—and given a feature value of 0—if it is not. We use rectangular receptive fields (tiles) of both 2x2 and 3x3, shifted by 1 in both x and y coordinates as well as iterated over the available actions. For a more detailed treatment of linear function approximation and coarse coding methods, see chapter 9 of Sutton and Barto.[33]

Hierarchical state space

The hierarchical state space experiments took place in the same gridworld environment and conditions as with the non-hierarchical (flat) learners. The difference was that the Q-learning policy that the agent learned was in relation to a different state space. Instead of the 118 grid states a 944 state-action pairs, this regional state space contained 10 states (regional groupings of grid states, e.g. the obstacle edge areas) and 40 state-action pairs (e.g. go to the shelter area from the left obstacle edge area). During the training phase, the agent’s policy was updated with respect to its transitions between these regions. For example, it would only update the value of its "go to the shelter area from the left obstacle edge area" immediately after crossing the border between those regions. Here, the distance function $d(s,s')$ that determines negative reward per timestep was equal to the distance between the centroids of the regions

that the agent moved between. When the agent executes its policy at test time, it produces high-level actions. To carry out these actions, its low-level controller simply carries out an innate ability to move directly in a straight line from its current position (e.g. the threat zone) to the target location (e.g. obstacle edge area), similar to Edvardsen et al.[36] We set up this hierarchical state space to use with Q-learning out of convenience, but it could have been used with the other gradual learners as well.

Successor Representation

The SR agent uses a model-free update rule to learn a representation of how state-action pairs predict (temporally discounted) future occupancy in each state in the environment. This successor representation, M, is thus a $SxAxS'$ tensor, where the index of the first two dimensions identifies a state-action pair and the third dimension corresponds to the successor state. M can be combined with a separately learned reward vector R in order to compute value:

$$Q(S,A) = \sum_{S'} M(S,A,s')R(s')$$

This equation shows that the value of a state-action pair is the product of how much that state-action pair predicts future occupancy in the rewarded states and how much reward is those states. In our experiments, there is at most one rewarded state, so this reduces to:

$$Q(S,A) = M(S,A,shelter)R(shelter)$$

In order to learn the successor representation M, the agent applies a model-free updating rule with an eligibility trace[64] to an entire row after each step:

$$M(s_t,a_t,:) \leftarrow M(s_t,a_t,:) + \alpha\left[1_{s_{t+1}} + \gamma E_a[M(s_{t+1},a,:)] - M(s_t,a_t,:)\right]e$$

where α is the learning rate, $1_{s_{t+1}}$ is a one-hot vector with a 1 in the position of the successor state s', γ is the temporal discount factor, e is the eligibility trace updated similarly to Q-learning as described above, and $E_a[\ldots]$ is the expected row in the SR for the successor state s', averaged across the possible actions taken from that state. SR values are initialized randomly with mean 0 and variance 1. Simultaneously, a reward vector must be learned. It is updated after each step:

$$R(s_t) \leftarrow R(s_t) + \alpha(r_t - R(s_t))$$

The reward vector is initialized to all zeros. Note that a non-zero reward initialization (along with a small learning rate) will cause the SR model to fail to find routes to shelter in condition 2 until the agent enters and exits the shelter multiple times.

Model-based agent

The model-based agent builds up a model of the environment in the form of an undirected graph. Each time the agent encounters a new state, it stores that state as a node in the graph. Each time the agent receives a reward, it labels the node from which the reward emanated with the amount of reward. Each time the agent takes a new transition between nodes, it stores that transition as an edge in the graph. Each time the agent attempts to make a transition and is blocked by an obstacle or trip wire, it deletes that edge from the graph. The immediate learner plans using the most recent set of edges. The gradual learner stores a buffer of up to N observations per edge. During planning, edges are only used if the majority of observations in the buffer indicate that the edge is not blocked. In addition, the reward in each state is taken to be the average reward observed over the past N observations. At decision time, the model-based agent uses its model to plan the shortest possible route to the reward location, where horizontal and vertical edges have a path length of 1.0 and diagonal edges have a path length of $\sqrt{2}$. This is a heuristic that maximizes the expected future reward in this navigation-task setting. Shortest routes were calculated using an A-star tree search algorithm.[65] Equally effective actions (according to the A-star algorithm, which finds the shortest route to the goal) were sampled with equal probability.

Practice runs

We augmented the random exploration policy during the training phase with practice edge-vector and shelter-vector runs. Edge-vector runs were hard-coded action trajectories taking the agent from the threat area directly to an obstacle edge. The initiation and termination states are shown in Figure 5. Each time the agent entered one of these states, the hard-coded trajectory was triggered with a probability of 0.2.

Classifying escape runs

We used four classifications for simulated escape runs: homing-vector routes, edge-vector routes, tortuous routes and non-escapes. Homing-vector routes went from the threat zone to one of the three middle states above the obstacle location, and then continued toward the shelter (south, southwest or southeast) from there. Edge-vector routes went from the threat zone to the obstacle edge, without deviating from its path by more than one step to go around the trip wire. Tortuous routes are homing-vector or edge-vector routes that deviate from that path (to go around a trip wire location) by at least two steps. Non-escapes did not reach the shelter within the 50-step time limit.

Neuron, Volume *111*

Supplemental information

Mice identify subgoal locations through an action-driven mapping process

Philip Shamash, Sebastian Lee, Andrew M. Saxe, and Tiago Branco

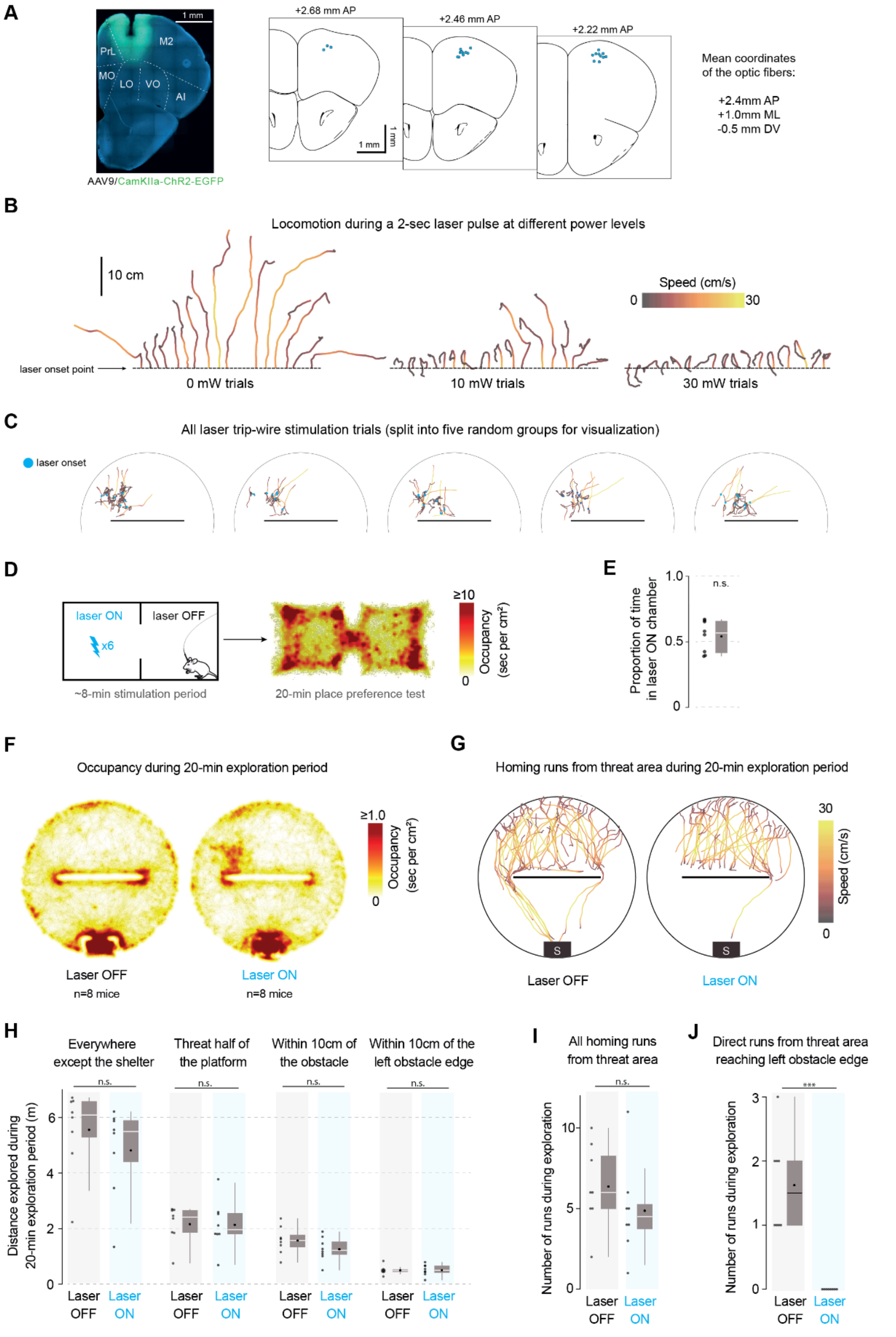

Figure S1: Optogenetic stimulation of right premotor cortex, Related to Figure 1

(A) Left: example viral injection and optic fiber implantation site. M2: supplementary motor cortex (premotor cortex), PrL: prelimbic cortex, MO/LO/VO: medial/lateral/ventral orbital cortex, AI: agranular insular cortex. Right: Putative optic fiber tip locations are overlaid on brain-slice diagrams adapted from [S1] Histological slice images were registered to the Allen Mouse Brain Atlas [S2] using SHARP-Track [S3]to find the fiber tip coordinates. The site of injection with channelrhodopsin was 0.25 mm below (ventral to) the fiber tip. AP and ML coordinates are relative to bregma, and DV coordinates are relative to the brain surface. **(B)** Locomotion following a 2-sec,

20-Hz, 30-mW pulse wave (duty cycle 50%) of 473-nm light in implanted mice. Laser stimulation was triggered manually upon initiation of a running bout, in the behavioral platform with no obstacle and no shelter. Each mouse received 4 trials at each laser power, sequentially interleaved. n = 4 mice. Lines are ordered by the distance and direction of movement following laser onset. **(C)** Trajectories before and after laser stimulation, for the edge-vector blocking protocol. n = 8 mice, 3.5 [2.75,6] (median [IQR]) laser stimulation trials per mouse. **(D)** Place preference assay. Each chamber in the place-preference arena (18 cm x 18 cm x 18 cm) has a distinguishing landmark on the back wall (a large cross and a large circle). After a 1-min habituation period, stimulation consisted of six trials of four repeated 2-sec, 20-Hz, 30-mW pulses (24 total pulses). Stimulation was manually triggered when the mouse fully entered the stimulation side, with at least one minute between trials. The side of stimulation was pseudo-randomly selected such that half of the mice were stimulated on each side. For the occupancy heatmap, stimulation is shown as if it were on the left side for all mice. The heatmap was smoothed with a gaussian filter (σ = 0.3 cm). **(E)** Occupancy in the stimulation chamber is not significantly below 50%. p = 0.7, one-tailed Wilcoxon signed-rank test. n = 8 mice. **(F)** Occupancy heatmaps, smoothed with a gaussian filter (σ = 1 cm). **(G)** Runs from all eight mice in each condition. Left: same runs as in Figure 1a, except with non edge-vector runs also included. Right: Homing runs do not reach the left obstacle edge due to the closed-loop optogenetic stimulation. **(H)** Distance explored is used instead of time explored to account specifically for active exploration. Everywhere except the shelter: p = 0.2; threat half: p = 0.5; obstacle: p = 0.1, edge: p = 0.5, one-tailed permutation tests. **(I)** Total number of homing runs (trajectories shown in panel b): p = 0.15. **(J)** Runs reaching the left edge: p = 3×10^{-5}, one-tailed permutation tests.

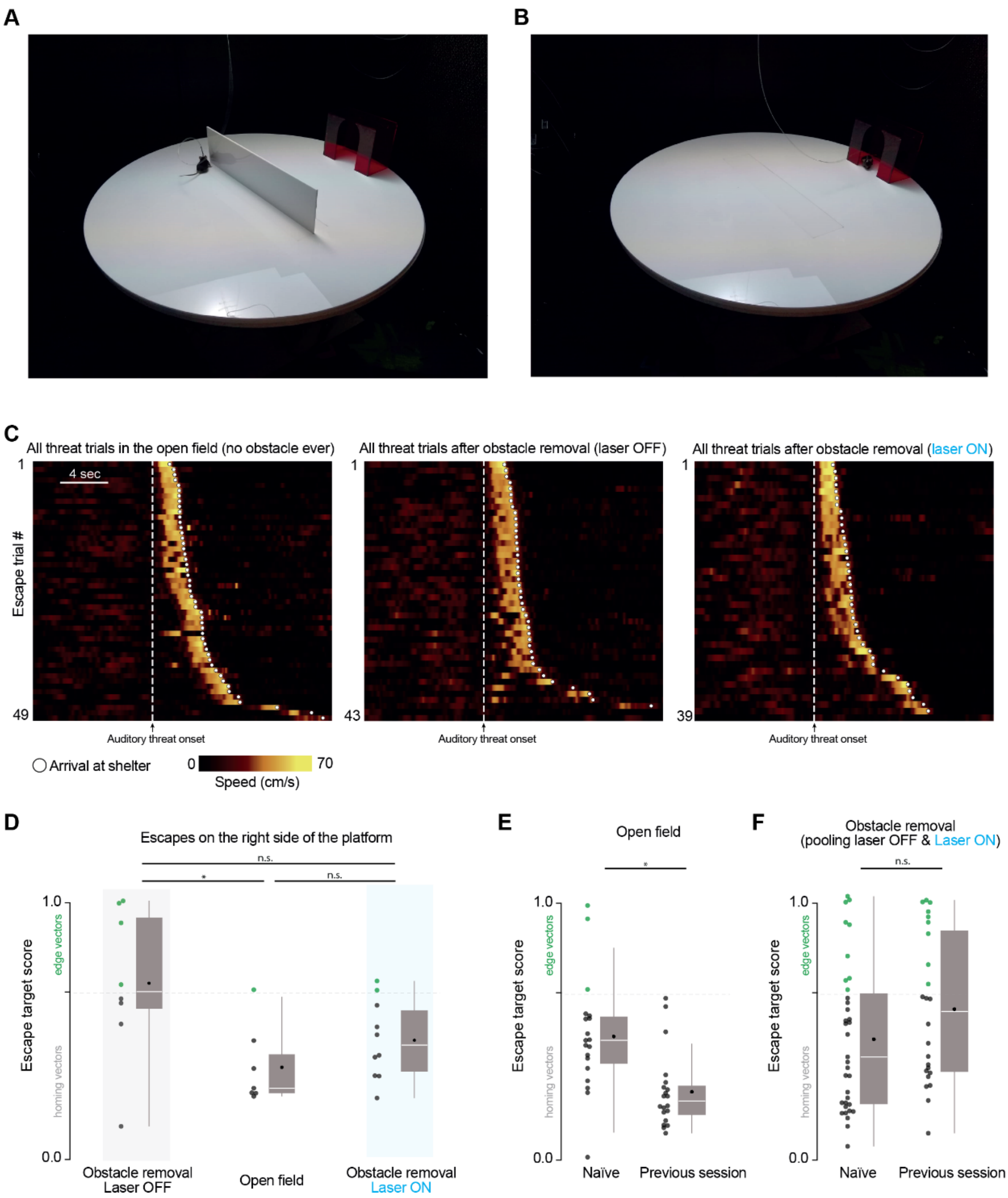

Figure S2: Escape behavior for different conditions, Related to Figure 2

(A) The platform with the wall obstacle. The platform is 92 cm in diameter, and the wall obstacle is 50 cm long x 12.5 cm tall. The shelter is 20 cm wide x 10 cm deep x 15 cm tall. It is made from red acrylic that is opaque to the mouse but transparent to red and infrared light. The mouse has is at the right obstacle edge. **(B)** The platform with no obstacle. A central panel (50 cm wide x 10 cm wide) with the obstacle has been replaced, and a flat panel has been slotted in, in its place. The mouse is sitting in the shelter. **(C)** Open field: n = 8 mice, 29 trials; obstacle removal (laser off): n = 8 mice, 26 trials; obstacle removal (laser on): n = 8 mice, 23 trials. Speed is smoothed with a gaussian filter (σ = 100 ms). **(D)** Escapes on the right side are defined as escapes that, upon passing the center of the arena along the shelter-threat (north-south) axis, were on the right half of the platform. In the main figures, we limited analysis to escapes on the left side of the platform (measured at the center of the shelter-threat axis). This allows us to evaluate our model, in which edge-vector runs generate subgoals one at a time, at the targeted edge. We observed that there were fewer escapes on the right side across all conditions, possibly due to an environmental bias or an effect of the brain implant. As a result, there are not enough data determine whether the laser manipulations has an effect on right-side escapes. Obstacle removal (laser off) vs. open

field: p = .045; Obstacle removal (laser on) vs. open field: p = .1; Obstacle removal (laser off) vs. obstacle removal (laser on): p = .1, one-tailed permutation tests on proportion of edge-vector escapes. Open field: 10 escapes; Obstacle removal (laser off): 8 escapes; Obstacle removal (laser on): 10 escapes. **(E)** In each experiment, 4/8 mice were naive, and 4/8 mice had had a previous behavioral session, in a random condition. Mice with a previous session targeted the shelter more accurately in the open-field environment. P = .03, one-tailed permutation test on proportion of edge-vector escapes. **(F)** Mice with a previous session did not execute significantly more edge-vector escapes than naive mice. p = 0.2, one-tailed permutation test on proportion of edge-vector escapes.

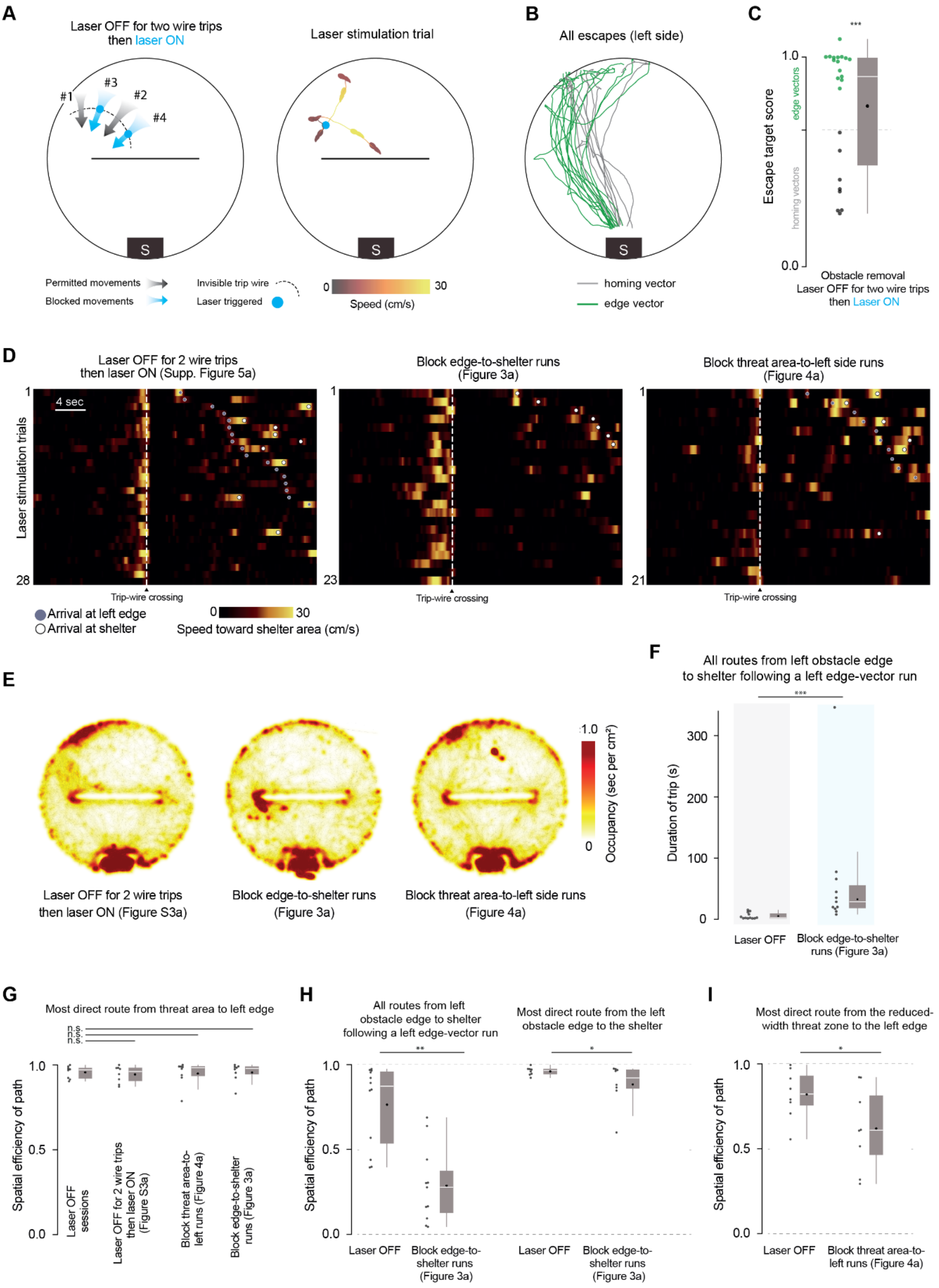

Figure S3: Controls for the effects of optogenetic stimulation, Related to Figure 3

(A) Schematic of stimulation blocking all but the first two edge-vector runs. Stimulation protocol follows the edge-vector-blocking protocol from Figure 1, except that the first two trip-wire crossings are allowed to occur with no laser stimulation. This generated a median of 3.0 laser-stimulation trials per mouse, compared to 3.5 in the original experiment from Figures 1-2. The example shows four seconds after laser onset: the mouse was stimulated for two seconds, and then ran toward the

center of the obstacle. **(B)** Escapes after obstacle removal; n = 8 mice, 23 escapes. **(C)** Obstacle removal (block after two crossings) vs. open field: $p = 3\times10^{-4}$ (***); vs. obstacle removal (block edge vectors): p=.003; vs. obstacle removal (laser off): p = .9, one-tailed permutation tests on proportion of edge-vector escapes. **(D)** All trip-wire crossings, with laser stimulation. Left, center: mice must be moving toward the shelter area (i.e., southward) in order to trigger the trip wire. Right: mice must be moving toward the left side in order to trigger the trip wire. **(E)** Occupancy heatmaps are smoothed with a gaussian filter (σ = 1 cm) and overlaid on all movements for all mice (transparent gray dots). There is increased occupancy in the north-west area of the platform in the laser-off-then-on; block threat-to-left-side; and original block-edge-vector conditions due to a pinch of bedding being placed in that area (see Methods). There is increased occupancy near the left obstacle edge in the block edge-to-shelter condition due to the optogenetic stimulation taking place there. **(F)** Each dot represents one trip between the left obstacle edge and the shelter. Laser off median: 2.5 sec; Block edge-to-shelter median: 29 sec. Block edge-to-shelter vs. laser off: p = 0.001, one-tailed permutation test. **(G)** Each dot represents once session (the most efficient path that took place during that session). Laser off-then-on vs. laser off: p = 0.4; block threat-to-left-side vs. laser off: p = 0.3; block edge-to-shelter vs. laser off: p = 0.2, one-tailed permutation tests. **(H)** Left: Each dot represents one trip between the left obstacle edge and the shelter; p = 0.002, one-tailed permutation test. Right: each dot represents one session; p = 0.02, one-tailed permutation test. The effect on the most direct route in the session is relatively weak; this is because, in this experiment, we only blocked edge-to-shelter movements that followed edge-vector runs (i.e., passed the original trip wire from Figure 1). Thus, edge-to-shelter movements that did not pass through the threat area (e.g., running from the shelter to the edge and back) were spared. **(I)** Each dot represents one session; p = 0.02, one-tailed permutation test.

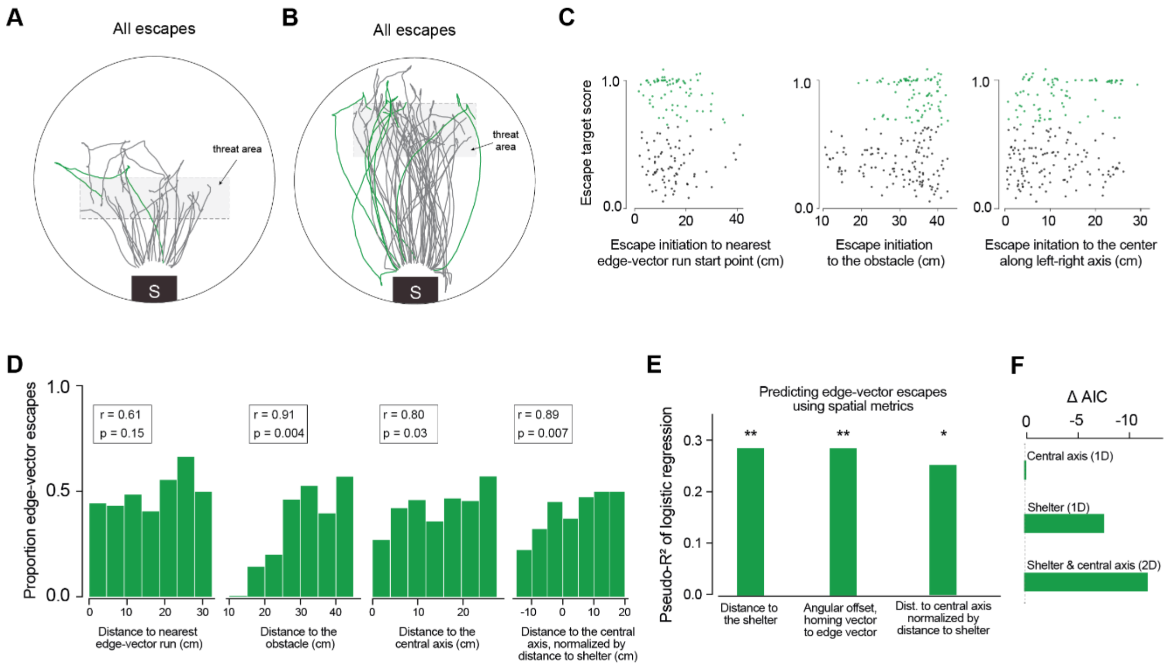

Figure S4: Relationship between escape onset location and subgoal behavior, Related to Figure 4

(A) Escapes triggered after obstacle removal, using the new threat zone, indicated by the red dotted lines. Green trace shows the only escape that begins by moving toward the obstacle edge location (1/28); however, this appears to be a continuation of the pre-threat movement rather than a genuine subgoal escape. **(B)** Escapes triggered after obstacle removal, using another new threat zone, indicated by the red dotted lines. **(C)** Distance metrics plotted against the escape target score. Edge-vector escapes are plotted in green. Distances are measured from the escape initiation point. For the distance to the nearest spontaneous edge-vector run start point (top), only runs toward the same side as the escape are considered. **(D)** To visualize the relationship between position and edge-vector probability, each spatial metric is put into seven equal-sized bins, and the proportion of edge-vector escapes in each bin is taken. All bins have at least six escapes. r-values and p-values come from the Pearson correlation between the spatial bin and the proportion of edge-vector escapes. **(E)** Since the metrics we tested are correlated with each other, we analyzed whether their relationship to escape behavior could all be parsimoniously explained by a sense of distance from the shelter. To test this, we normalized the mouse's distance from the central axis such that it was uncorrelated to the distance from the shelter, using linear regression. This normalized spatial metric retained its capacity to predict edge-vector escapes (pseudo-R^2=0.25; p=0.017, permutation test). This means that, at a given distance from the shelter, mice are more likely to execute edge-vector escapes if they are further from the central axis of the arena McFadden's pseudo-R^2 measures the strength of the relationship between each metric and the odds of executing edge-vector escapes. Distances and angles are measured from the escape initiation point of each escape. For the distance to the nearest spontaneous edge-vector run start point, only runs toward the same side as the escape are considered. Distance to the shelter: pseudo-R^2=0.29; p=0.006. Angular offset between the homing vector and the edge vector: pseudo-R^2=0.29; p=0.006. Distance to the central axis, normalized by the distance to the shelter: pseudo-R^2=0.25; p=0.017. P-values come from a permutation test using 10,000 random shuffles of the edge-vector/homing-vector labels, with the pseudo-R^2 as the test statistic. **(F)** Akaike Information Criterion (AIC) analysis on a logistic regression with different predictors. Decreases in AIC represent better model fit and include a penalty for using additional parameters/predictors (with substantially better fit at δAIC <=2 [S4]). $\delta AIC_i = AIC_i \quad AIC_{m}in$ where $AIC_{m}in$ here is the AIC from the model with the single, distance-from-central-axis predictor.

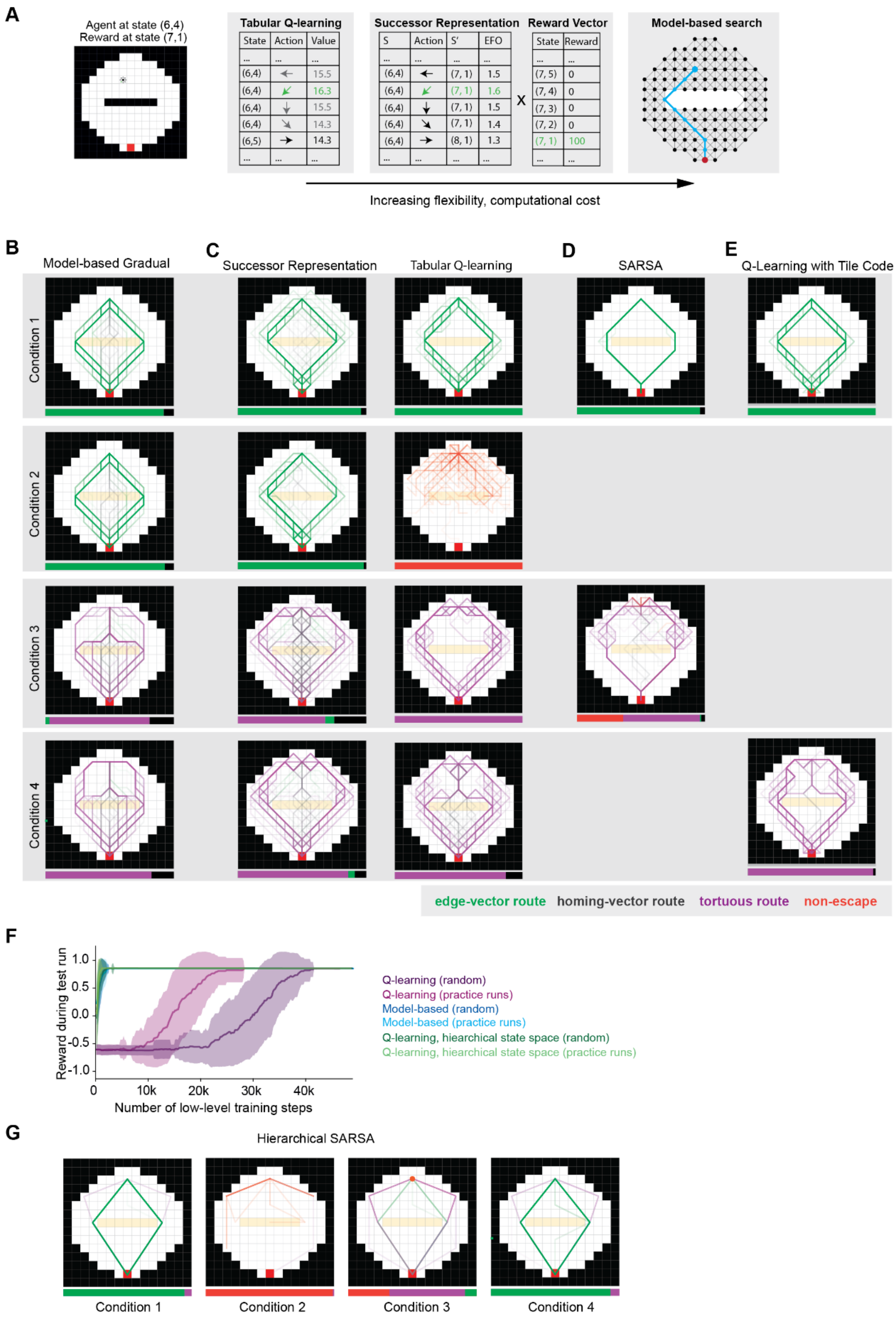

Figure S5: Reinforcement learning models of mouse escape behavior, Related to Figure 5

(A) Schematic of the core reinforcement learning models that we used. Q-learning updates a table of values for each state-action pair. Successor Representation updates a matrix recording how much each state predicts future occupancy in each other state, as well as a table of how much reward is in each state. Our model-based agents update a list of states, a graph of connections between each state, and a label of the amount of reward in each state. **(B)** Results for the gradual-

learning model-based agent. This agent uses the past 15 observations of each edge in the graph to decide if two adjacent states are connected or blocked by a barrier. Escape runs from all three trials of all 100 random seeds in all four conditions. All trials are superimposed, with high transparency. Bars under each plot show the proportion of each type of escape. **(C)** Results for the successor representation and Q learning, with random exploration (no practice runs). **(D)** Results for the SARSA agent. This agent is similar to tabular Q-learning, but it learns *on-policy*, meaning that the policy it converges to depends on its exploration policy. **(E)** Results for Q-learning with tile coding. Instead of a representing each state individually, tile-coding agent represents its approximate location with a set of n x n tiles (Methods). We used the coarsest-grained features with which the agent was still able to find a route to shelter was used (both 2x2 and 3x3 tiles, at once). **(F)** Learning curves plot the amount of reward received from a trial run in the test map (going from threat zone to shelter) over number of training steps. For the learning curves, there is no pre-test phase; the agent is simply dropped in the threat zone. The trial terminates when the agent reaches the shelter or at a maximum number of 50 steps. Since negative reward is given at each non-reward state (Methods), the minimum reward is approximately -0.5. **(G)** Results with the hierarchical state-space agent using the SARSA algorithm, with practice runs during exploration. Assuming a homing-vector agent takes over when this agent fails to come up with a route, this agent qualitatively matches mouse behavior on all four conditions: edge vectors in condition 1 and 4 and failure to learn a route in conditions 2 and 3. The orange dot in condition 3 indicates selecting an invalid action.

Supplemental References

S1 - Paxinos, G., & Franklin, K. B. J. (2019). *Paxinos and franklin's the mouse brain in stereotaxic coordinates*. Academic Press.

S2 - Allen Institute for Brain Science. (2015). Allen mouse brain atlas. https://mouse.brain-map.org/static/ atlas

S3 - Shamash, P., Carandini, M., Harris, K., & Steinmetz, N. (2018). A tool for analyzing electrode tracks from slice histology. *bioRxiv*.

S4 - Burnham, K. P., & Anderson, D. R. (2004). Multimodel inference. Sociol. Methods Res., 33(2), 261–304.
